# Centers performance for end-stage kidney replacement therapy: a Bayesian hierarchical logistic regression for a binary kidney transplant status

**DOI:** 10.1186/s12874-026-02800-9

**Published:** 2026-02-20

**Authors:** Solomon Woldeyohannes, Alan Cass, Yomei Jones, Paul Lawton

**Affiliations:** 1 Menzies School of Health Research, Charles Darwin University, Casuarina, Darwin, 0811 Northern Territory Australia; 2 School of Veterinary Science, University of Queensland, Main Dr & Outer Ring Rd, Gatton, 4343 Queensland Australia

**Keywords:** Bayesian inference, Gibbs sampling, Metropolis-Hastings, Hierarchical logistic regression, Kidney transplant prediction, Centre profiling

## Abstract

**Purpose:**

The past two decades witnessed increased use of risk-adjusted standardized measures such as standardized mortality ratios (SMRs) and standardized incidence ratios (SIRs) in institutional comparisons between healthcare units. Estimating the variance of these standardised measures is necessary for creating false discovery rates (FDRs) in studies that use funnel plots for assessing healthcare providers’ performance. Theoretical delta-method, and approximate approaches such as bootstrapping and Bayesian approaches are commonly used. Using Bayesian hierarchical logistic regression for obtaining estimated standardised performance measure, introduces non-conjugacy in the posterior distribution of the fixed and random effects parameters. The non-conjugate parameters require Metropolis-Hastings (MH) steps within the Gibbs sampling algorithm. For this, JAGS and BUGS software are used to specify prior distribution and run sampling. While JAGS and Stan are flexible and reduce programming burden, manual control of the sampling algorithm, can offer advantages in fine-tuning the sampling process, especially for complex hierarchical models or when exploring alternative priors.

**Methods and analysis:**

Posterior computation of intractable distributions involves deriving and sampling from full conditionals of model parameters and hyperparameters. Therefore, we derived full conditionals of the parameters of a Bayesian hierarchical logistic regression model for a binary kidney transplant status across centers in Australia and apply MH steps. Our model includes centre-level random intercepts and patient-level covariates that spans from 2006 to 2023. Model based predicted probabilities were used to estimate expected kidney transplant counts per centre and log-standardised incidence ratios were used as performance measures for classifying centers.

**Results:**

We found posterior point estimates and uncertainty summaries highly consistent across the MH, R2jags, and Stan implementations, indicating that substantive inference is robust to the choice of sampling algorithm and software platform. Our finding indicated that, stable posterior estimates can be achieved (PSRF $\approx 1.0$ and ESS $> 200$) for the regression parameters but for covariates with sparse data.

**Conclusion:**

The results support the conclusion that the custom MH implementation provides valid and reliable posterior inference for the present application despite the reduced computational efficiency for certain parameters relative to R2jags and Stan. It also reinforces its use in stabilizing centers with limited patient volume.

## Introduction

Assessing health providers’ performance in the healthcare system has been an area of interest over the past three decades [1, 2]. Hierarchical logistic regression modeling for binary outcome variables is commonly employed to adjust for patient case-mix and centre-level covariates [3, 4]. Standardized mortality ratios (SMRs) or standardized incidence ratios (SIRs) are then computed as comparative measures of providers’ performance for specific outcomes. League tables, forest plots, and more recently, funnel plots are commonly used to visualize the relative performance of providers [5, 6].

To quantify uncertainty in SMR/SIR estimates, resampling methods such as bootstrapping are often employed to generate confidence intervals (CIs) or false discovery rates (FDRs) around point estimates [7]. Despite the computational intensity, Bayesian approaches have been increasingly recommended for assessing centre performance due to their ability to provide coherent uncertainty quantification and to naturally incorporate hierarchical structures [8, 9].

One notable advantage of Bayesian approaches is their ability to appropriately handle centers with small patient volumes [10], which is critical in preventing over-interpretation of unstable estimates [11]. Bayesian hierarchical logistic regression (BHLR) remains a prominent tool for evaluating hospital and provider performance in health services research due to its ability to account for multilevel data structures and uncertainty in parameter estimates [12].

Practical usage of Bayesian hierarchical logistic regression models for healthcare performance evaluation include adjusting patient risk [12], controlling for provider-level variability via shrinkage [13], including low-volume providers [10], and enabling decision-theoretic classifications via posterior probabilities [14]. Austin (2008) developed Bayesian hierarchical logistic regression models for hospital profiling, modeling patient-level mortality $p_{j[i]}$ with hospital-specific random effects for intercepts (and slopes). They derived decision rules under different loss functions to classify hospitals with unacceptable mortality. Bayesian rules help reduce regression to the mean and facilitate the inclusion of low-volume hospitals [13]. Yang et al (2013) using California coronary artery bypass grafting (CABG) data, compared Bayesian and frequentist hierarchical logistic regression models via simulations. They found hierarchical (Bayesian) methods are more conservative-fewer false outliers-though may exhibit over-shrinkage, providing theoretical and empirical guidance for profiling under varying sample sizes [10]. Normand et al (2007) review outlines hierarchical logistic regression methods for hospital profiling, addressing risk adjustment, shrinkage priors, and shrinkage in estimating hospital-specific effects, while highlighting Bayesian alternatives and challenges like small volume hospitals [12].

Accurate modeling of patient outcomes across hospitals/centers is critical for health system evaluation and improvement [1, 2]. Traditional frequentist methods may face challenges when incorporating hierarchical structures and quantifying uncertainty, particularly in settings with small sample sizes or unbalanced data [3, 4]. Bayesian hierarchical models offer a natural framework for addressing these complexities [8, 9], and Gibbs sampling provides a computationally tractable approach to inference in high-dimensional posterior spaces [15]. A critical aspect of implementing BHLR is the choice of computational algorithm for posterior inference. In hierarchical logistic regression, the presence of a logistic link introduces non-conjugacy in the posterior distribution of the fixed and random effects parameters, particularly when dealing with binary outcomes. While Gibbs sampling is straightforward for parameters with conjugate priors (e.g., normally distributed random effects with normal priors), non-conjugate parameters require Metropolis-Hastings (MH) steps within the Gibbs sampling algorithm. Chapter 16 in Gelman et al (2013) discusses hierarchical models and computation, including the need for Metropolis steps when dealing with non-conjugate posteriors in logistic regression [15]. When full conditional distributions are not available in closed form, Metropolis-Hastings (MH) steps within Gibbs sampling become essential to enable sampling from complex posterior distributions [16, 17].

Within the Bayesian paradigm, R packages such as R2jags, JAGS, and BUGS facilitate posterior sampling by generating samples from a proposal equilibrium distribution that, under convergence, represents the posterior distribution as function of $\theta$, the parameter of interest.

In this work, full conditionals for the parameters of a Bayesian hierarchical binary logistic regression model were derived and a Gibbs sampler via Metropolis-Hastings (MH) accept-reject approach was proposed for sampling from the full conditionals of the model. While standard R packages such as R2jags and Stan are available for running the analysis, we initially choose the custom MH implementation for pedagogical transparency and to allow custom sampling of variance components under alternative prior specifications. R2jags leverages the JAGS engine, which combines Gibbs sampling with randomly selected Metropolis updates for non-conjugate parts of the model. Gibbs sampling can be very efficient for models with conditional conjugacy, but in logistic models without a convenient full conditional, R2jags effectively uses MH or slice updates under the hood. Although R2jags simplifies model specification and adaptation, but its samplers remain local update methods that may struggle with strong correlations or hierarchical dependencies [18].

In contrast, Stan uses HMC/NUTS, which exploits gradient information of the posterior to make informed global proposals. HMC avoids the random-walk behavior of MH by simulating Hamiltonian dynamics, substantially reducing autocorrelation and improving effective sample size per unit of computation [19, 20]. NUTS further automates the choice of trajectory length, making Stan’s implementation of HMC highly robust for a wide class of models. However, we acknowledge that this approach introduces tuning challenges and slower mixing relative to HMC [15, 16]. The Metropolis–Hastings (MH) algorithm is one of the earliest Markov chain Monte Carlo (MCMC) methods, relying on proposal distributions and acceptance probabilities to explore the posterior. Its simplicity is appealing, but its performance heavily depends on carefully chosen proposal scales and acceptance rates; poorly tuned proposals can lead to slow exploration and high autocorrelation, especially in high-dimensional or correlated parameter spaces. MH is a random-walk sampler without gradient information, making it particularly prone to slow mixing in hierarchical models with complex posterior geometry [16]. To address this concern empirically, we added a comparative analysis in the discussion comparing our sampler to a Stan and JAGS implementations of the same model, reporting effective sample size per second and runtime.

The model is formulated with centre-specific random intercepts for modeling patients’ kidney transplant status at two years starting kidney replacement therapy (KRT). It includes key predictors such as age group, gender, Indigenous status, lung disease, diabetes, obesity, cardio-vascular diseases (CVD), referral status, remoteness and time period. We implemented a hybrid Gibbs-MH algorithm to sample from the posterior, enabling full Bayesian inference on both individual and centre-level effects.

The rest of the paper is organised as follows. Firstly, we present fundamental theoretical results that justify the steps in deriving full conditional distributions for a Bayesian hierarchical logistic regression model. Secondly, we derive the full conditionals for the respective parameters. Finally, model diagnostics, results and discussion of the key findings are presented.

## Methods

This section is started with the fundamental Bayes’ theorem and the law of total probability, which are used to underpin the derivation of the full conditionals for the parameters and hyperparameters of a Bayesian hierarchical logistic regression model.

### Bayes’ theorem

#### Theorem 1

Given data $Y$ and parameters $\theta$, the posterior distribution is proportional to the product of the likelihood and the prior:

$$\begin{aligned} P(\theta \mid Y) \propto P(Y \mid \theta ) P(\theta ) \end{aligned}$$

This theorem underpins the derivation of all full conditionals in Bayesian inference [15].

### Law of total probability and marginalization

#### Theorem 2

Let $\theta = (\theta _1, \theta _2)$. Then the marginal posterior for $\theta _1$ is:

$$\begin{aligned} P(\theta _1 \mid Y) = \int P(\theta _1, \theta _2 \mid Y) \, d\theta _2 \end{aligned}$$

#### Corollary 1

This marginalization principle guides the derivation of full conditional distributions by conditioning on all other parameters [15].

The above mariginalization principle can easily be expanded for models involving more than two parameters ($p > 2$) as indicated in the next theorem.

#### Theorem 3

Let $\boldsymbol{\theta } = (\theta _1, \theta _2, \ldots , \theta _p)$. Then the marginal posterior distribution for a single parameter $\theta _j$, for any $j \in \{1, \dots , p\}$, is obtained by integrating the joint posterior over all other parameters:

$$\begin{aligned} P(\theta _j \mid Y) = \int \cdots \int P(\theta _1, \ldots , \theta _p \mid Y) \, d\theta _1 \cdots d\theta _{j-1} \, d\theta _{j+1} \cdots d\theta _p \end{aligned}$$

### Model specification

Let $y_{j[i]}$ denote the binary kidney transplant outcome for patient *i* in centre *j*, where $y_{j[i]} \in \{0,1\}$. Let $\textbf{x}_{j[i]}$ be the vector of predictors.

The hierarchical logistic regression model is formulated using the following logit link function:

1 $$\begin{aligned} \text {logit}(P(y_{j[i]}=1)) = \eta _i = \alpha + \textbf{x}_{j[i]}^T \boldsymbol{\beta } + u_j, \end{aligned}$$

where $\boldsymbol{\beta }$ are fixed effects, and $u_j \sim \mathcal {N}(0, \sigma ^2_u)$ are center-level random intercepts.

The priors and hyperpriors of the model parameters are specified using the normal distribution as follows: $$\alpha \sim N(0,\sigma _\alpha ^2)$$, $$\beta \sim N(0,\Sigma _\beta )$$, $$u_j \sim N(0,\sigma _u^2)$$, and $$\sigma _u^2 \sim \text {IG}(a_0, b_0)$$. The hyperparameters were specified as $$a_0 = 1.0$$ and $$b_0 = 1.0 $$.

For the between-hospital variance $\sigma _u^2$, we used $\sigma _u^2 \sim \text {Inv-Gamma}(1.0, 1.0)$, following traditional Bayesian practice for variance components in hierarchical models [21, 22].

While recent literature favors more informative priors for variance parameters [23, 24], the Inverse-Gamma(1,1) prior maintains conjugacy with the normal likelihood for random effects, enabling the Gibbs update. Specifically, conditional on the hospital-level random effects $u = (u_1, \dots , u_j)$, the full conditional distribution of the between-hospital variance is given by:

2 $$\begin{aligned} \sigma _u^2 \mid \textbf{u} \sim \text {Inv-Gamma}\left( a_0 + \frac{J}{2}, b_0 + \frac{1}{2}\sum \limits _{j=1}^J u_j^2\right) \end{aligned}$$

as described by Geman and Geman [25]. Where $$a_0 = 1, b_0 = 1 $$ are the hyperparametrs for $$\sigma ^2_u$$.

### Posterior sampling

The full joint posterior is proportional to:

3 $$\begin{aligned} P(\alpha,\boldsymbol{\beta},\mathbf{u},\sigma_u|\mathbf y) \propto \left( \prod_{j[i]} P(y_i|\alpha,\boldsymbol{\beta},u_{j[i]}) \right) P(\alpha) P(\boldsymbol{\beta}) \left( \prod_j P(u_j|\sigma_u) \right) P(\sigma_u) \end{aligned}$$

We use Gibbs sampling where each parameter is updated from its full conditional. For parameters without closed-form conditionals (e.g., $\boldsymbol{\beta }$), we employ MH steps.

### Exponential family and non-conjugacy of the joint posterior distribution

#### Theorem 4

The logistic likelihood function belongs to the exponential family [26]. However, when combined with Gaussian priors on the parameters $\alpha , \beta , u$, the posterior distribution is non-conjugate and does not belong to any known family of standard distributions.

#### Corollary 2

Posterior distributions for logistic models with Gaussian priors must be approximated using numerical methods, such as Metropolis-Hastings or Hamiltonian Monte Carlo [15].

#### Proof of posterior non-conjugacy

We have, the likelihood for a binary outcome $Y_i \sim \text {Bernoulli}(\sigma (\eta _i))$ where $\eta _i = \alpha + X_i^\top \beta + u_{j[i]}$, $\text {with P(Y}_i=1) = \sigma (\eta _i)$, and priors for the parameters as $\alpha \sim N(0,\sigma _\alpha ^2)$, $\beta \sim N(0,\Sigma _\beta )$ and $u \sim N(0,\sigma _u^2I)$. And following Eq. 2, therefore, the joint posterior distribution can be expressed as:

4 $$\begin{aligned} \boxed{ P(\alpha, \boldsymbol{\beta}, \mathbf{u}, \sigma_u \mid \mathbf{y}) \propto P(\mathbf{y} \mid \alpha, \boldsymbol{\beta}, \mathbf{u}) P(\alpha) P(\boldsymbol{\beta}) P(\mathbf{u} \mid \sigma_u) P(\sigma_u)} \end{aligned}$$

Substituting the likelihood and priors in Eq. (3), we get final expression of the joint posterior distribution shown in Eq. 4.

5 $$\begin{aligned} P(\alpha ,\beta ,u|Y) & \propto \left[ \prod _{i=1}^n \sigma (\eta _i)^{Y_i}(1-\sigma (\eta _i))^{1-Y_i}\right] \nonumber \\ & \times \exp \left\{ -\frac{1}{2\sigma _\alpha ^2}\alpha ^2\right\} \nonumber \\ & \times \exp \left\{ -\frac{1}{2}\beta ^\top \Sigma _\beta ^{-1}\beta \right\} \times \exp \left\{ -\frac{1}{2\sigma _u^2}\Vert u\Vert ^2\right\} \end{aligned}$$

Taking the logarithm of the above joint posterior, we have the log-posterior (dropping constants) as shown below:

6 $$\begin{aligned} \log P & = \sum \limits _{i=1}^n \left[ Y_i\eta _i - \log (1+e^{\eta _i})\right] - \frac{1}{2\sigma _\alpha ^2}\alpha ^2 \nonumber \\ & \quad - \frac{1}{2}\beta ^\top \Sigma _\beta ^{-1}\beta - \frac{1}{2\sigma _u^2}\Vert u\Vert ^2 \end{aligned}$$

Therefore, ite is evident that no combination of terms cancels out to form a known distribution and it is known that gaussian prior quadratic forms don’t combine nicely with the logistic likelihood to get closed-form [15, 27]. And it can be concluded that while the logistic likelihood is in the exponential family, its combination with Gaussian priors leads to a non-conjugate posterior requiring approximation methods (MCMC, variational inference, or Laplace approximation). This explains the need for computational methods in Bayesian logistic regression [27]. The non-conjugacy arises fundamentally from the incompatibility between the logistic cummulative distribution function (CDF) and the Gaussian density’s quadratic form [15]. Therefore, in the next subsections, we drived full conditionals for the respective parameters of the random effects logistic regression so that Gibbs sampling via MH is feasible.

#### Derivation of full conditionals and gradients for Metropolis-Hastings

From (1), the likelihood and priors for a binary outcome *Y* can be specified as follows: $Y_i \sim \text {Bernoulli}(\sigma (\eta _i))$ where $\eta _i = \alpha + X_i^\top \beta + u_{j[i]}$, $\alpha \sim N(0,\sigma _\alpha ^2) \ \beta \sim N(0,\Sigma _\beta ) \ u_j \sim N(0,\sigma _u^2) \ \sigma _u^2 \sim \text {IG}(a_0, b_0)$.

In the following subsections, the full conditionals for each of the parameters of the hierarchical logistic regression model have been derived.

##### Full Conditional for $\alpha$

By isolating terms containing $\alpha$ in quation (5), the full conditional for $\alpha$ becomes:

$$\begin{aligned} P(\alpha |\cdot )& \propto \left[ \prod _{i=1}^n \sigma (\eta _i)^{Y_i}(1-\sigma (\eta _i))^{1-Y_i}\right] \\&\exp \left\{ -\frac{\alpha ^2}{2\sigma _\alpha ^2}\right\} \end{aligned}$$

And the log-posterior (dropping constants) can be expressed as:

$$\begin{aligned}& \log P(\alpha |\cdot ) = \sum \limits _{i=1}^n \left[ Y_i\eta _i - \log (1+e^{\eta _i})\right] \\&- \frac{\alpha ^2}{2\sigma _\alpha ^2} \end{aligned}$$

Finally, the gradient for Metropolis-Hastings can be derived as:

$$\begin{aligned} \frac{\partial }{\partial \alpha }\log P(\alpha |\cdot ) = \sum \limits _{i=1}^n (Y_i - \sigma (\eta _i)) - \frac{\alpha }{\sigma _\alpha ^2} \end{aligned}$$

##### Full Conditional for $\beta$

Similarly, by isolating $\beta$ terms from Eq. (1), the full conditional for $\beta$ is derived as:

$$\begin{aligned}& P(\beta |\cdot ) \propto \left[ \prod _{i=1}^n \sigma (\eta _i)^{Y_i}(1-\sigma (\eta _i))^{1-Y_i}\right] \\&\exp \left\{ -\frac{1}{2}\beta ^\top \Sigma _\beta ^{-1}\beta \right\} \end{aligned}$$

With a log-posterior:

$$\begin{aligned} \log P(\beta |\cdot ) &= \sum \limits _{i=1}^n \left[ Y_i(X_i^\top \beta ) - \log (1+e^{\eta _i})\right] \\&- \frac{1}{2}\beta ^\top \Sigma _\beta ^{-1}\beta \end{aligned}$$

And gradient vector:

$$\begin{aligned} \nabla _\beta \log P(\beta |\cdot ) = \sum \limits _{i=1}^n (Y_i - \sigma (\eta _i))X_i - \Sigma _\beta ^{-1}\beta \end{aligned}$$

##### Full Conditional for $u_j$

Again here, for each centre *j*

$$\begin{aligned}& P(u_j|\cdot ) \propto \prod _{i:j[i]=j} \sigma (\eta _i)^{Y_i}(1-\sigma (\eta _i))^{1-Y_i} \\&\times \exp \left\{ -\frac{u_j^2}{2\sigma _u^2}\right\} \end{aligned}$$

The log-posterior

$$\begin{aligned} \log P(u_j|\cdot ) = \sum \limits _{i:j[i]=j} \left[ Y_i \eta _j - \log (1+e^{\eta _i})\right] - \frac{u_j^2}{2\sigma _u^2} \end{aligned}$$

And the gradient vector becomes:

$$\begin{aligned} \frac{\partial }{\partial u_j}\log P(u_j|\cdot ) = \sum \limits _{i:j[i]=j} (Y_i - \sigma (\eta _i)) - \frac{u_j}{\sigma _u^2} \end{aligned}$$

##### Full Conditional for $\sigma _u^2$

From Eq. (1), it is evident that only the prior and random effects depend on $\sigma _u^2$. Therefore, the full conditional for $\sigma ^2_u$ can be expressed as:

$$\begin{aligned} &P(\sigma _u^2|\cdot ) \propto \left[ \prod _{j=1}^J \frac{1}{\sqrt{2\pi \sigma _u^2}}e^{-\frac{u_j^2}{2\sigma _u^2}}\right]\\& \times \frac{b_0^{a_0}}{\Gamma (a_0)}(\sigma _u^2)^{-(a_0+1)}e^{-b_0/\sigma _u^2} \end{aligned}$$

And simplifying (see below the Normal-Inverse-Gamma Conjugacy for Random Effect Variance therom and derivation)

$$\begin{aligned} \sigma _u^2|\cdot \sim \text {IG}\left( a_0 + \frac{J}{2}, b_0 + \frac{1}{2}\sum \limits _{j=1}^J u_j^2\right) \end{aligned}$$

In the below, we employed an important result from the Normal-Inverse-Gamma Conjugacy for Random Effect Variance using the theorem along with its proof:

##### Theorem 5

(Normal-Inverse-Gamma Conjugacy) If $u_j \sim \mathcal {N}(0, \sigma _u^2)$ and $\sigma _u^2 \sim \text {IG}(a_0, b_0)$, then the posterior distribution is:

$$\begin{aligned} \sigma _u^2 \mid \{u_j\} \sim \text {IG} \left( a_0 + \frac{J}{2},\ b_0 + \frac{1}{2} \sum \limits _j u_j^2 \right) \end{aligned}$$

This result is derived by combining the Gaussian likelihood (for $u_j$) and the Inverse-Gamma prior using the rules of conjugate priors in exponential families [28]. For a complete proof of Normal-Inverse-Gamma conjugacy, see Appendix C.

#### MH Implementation

For $\alpha$, $\beta$, and $u_j$, we used MH and the gradients provided for efficient proposals. Also, for $\sigma _u^2$, since conjugate update combines prior with sum of squared random effects, direct sampling from Inverse-Gamma posterior was implemented. Details of the MH algorithm is presented in Appendix E.

### Decision rule for MH accept-reject step

Since the posterior does not have a closed form, we used the following accept-reject rule for each iteration during the MH sampling:

For $\alpha$, propose: $\alpha ^* \sim \mathcal {N}(\alpha , \tau _\alpha ^2)$ and accept with probability:

$$\begin{aligned}& \min \left( 1, \frac{L(\alpha ^*) p(\alpha ^*)}{L(\alpha ) p(\alpha )}\right) \\&= \min \left( 1, \exp (\log L(\alpha ^*) \right.\\&\left.- \log L(\alpha )\right. \quad \left.\right.\\&\left.+ \log p(\alpha ^*) - \log p(\alpha ))\right) \end{aligned}$$

where $L(\cdot )$ is the bernoulli likelihood evaluated at proposed/current $\alpha$.

For $\beta _k$, propose: $\beta _k^* \sim \mathcal {N}(\beta _k, \tau _\beta ^2)$ and use MH to accept/reject with probability:

$$\begin{aligned}& \min \left( 1, \frac{L(\beta ^*_k) p(\beta ^*_k)}{L(\beta _k) p(\beta _k)}\right) \\&= \min \left( 1, \exp (\log L(\beta _k^*) - \log L(\beta _k) \right. \\ & \quad \left. + \log p(\beta _k^*) - \log p(\beta _k))\right) \end{aligned}$$

Similarly, for $u_j$, we used MH:

Propose $u_j^* \sim \mathcal {N}(u_j, \tau _u^2)$ and compute the log-likelihood difference from patients in hospital *j* only. That is, the likelihood only depends on patients in hospital *j*, so only those data points are used. Hence, to update $u_j$, accept the proposed value $u_j^*$ with probability:

$$\begin{aligned} & \min \left( 1, \frac{L(u_j^*) p(u_j^*)}{L(u_j) p(u_j)}\right) \\ & = \min \left( 1, \exp (\log L_j(u_j^*) - \log L_j(u_j)\right. \\ & \quad \left. + \log p(u_j^*) - \log p(u_j))\right) \end{aligned}$$

### Predictions and computing the Standardised Incidence Ratio (SIR)

In the subsequent subsections, we have used the following definitions:

- $O_j = \sum \limits _{i:j[i]=j} Y_{j[i]}$ is the observed count patients receiving kidney transplant within two years of starting treatment at center *j*.
- $N_j$: number of patients at center $j$
- $E_j$: expected number of patients receiving kidney transplant within two years of starting treatment at center $j$ under the global model

#### Posterior predictive distribution

For each posterior sample *s* (from *S* MCMC samples), compute:

7 $$\begin{aligned} p_i^{(s)} = \sigma \left( \alpha ^{(s)} + X_i^\top \beta ^{(s)} + u_{j[i]}^{(s)}\right) \end{aligned}$$

where $\sigma (\cdot )$ is the logistic function.

#### Expected counts for each centre *j*

 

8 $$\begin{aligned} E_j = \sum \limits _{s=1}^S \sum \limits _{i:j[i]=j} p_i^{(s)} \end{aligned}$$

#### Standardized Incidence Ratio (SIR)

 

9 $$\begin{aligned} \text {SIR}_j = \frac{O_j}{E_j} \end{aligned}$$

where $E_j = \sum \nolimits _{i \in H_j} \hat{p}_i$, with $\hat{p}_i$ being the estimated probability of event for patient *i* in center *j*.

#### Implementation steps

For a summary of the implementation steps, refer to Appendix D. Computing Log Standardised Incidence Ration (Log-SIR).

1. Run MCMC to obtain posterior samples: $\{\alpha ^{(s)}, \beta ^{(s)}, u^{(s)}, \sigma _u^{2(s)}\}_{s=1}^S$
2. Compute probabilities for each observation:
  10 $$\begin{aligned} p_i^{(s)} = \frac{1}{1+\exp \left( -\left( \alpha ^{(s)} + X_i^\top \beta ^{(s)} + u_{j[i]}^{(s)}\right) \right) } \end{aligned}$$
3. Calculate expected counts:
  11 $$\begin{aligned} E_j^{(s)} = \sum \limits _{i:j[i]=j} p_i^{(s)} \end{aligned}$$
  then average across samples: $E_j = \frac{1}{S}\sum \nolimits _{s=1}^S E_j^{(s)}$
4. Compute SIR with credible intervals:
  Point estimate: $\text {SIR}_j = O_j/E_j$ and $\text {Log-SIR}_j = log(\text {SIR}_j)$
  95% Credible Interval (CrI): Use quantiles of $\{O_j/E_j^{(s)}\}_{s=1}^S$

### Funnel plots for comparing centers using Log-SIR

The funnel plot displays $\log (\text {SIR}_j)$ against a measure of precision ($1/\sqrt{E_j}$). The control limits at level $(1-\alpha )100\%$ are:

12 $$\begin{aligned} \text {Upper Limit} = \mu + z_{1-\alpha /2} \cdot \sigma _j \end{aligned}$$

13 $$\begin{aligned} \text {Lower Limit} = \mu - z_{1-\alpha /2} \cdot \sigma _j \end{aligned}$$

where $\mu$ is the overall mean (typically 0 since we used $\log (\text {SIR})$) for comparing the centers, $\sigma _j = \sqrt{\text {Var}[\log (\text {SIR}_j)]}$, and $z_{1-\alpha /2}$ is the $(1-\alpha /2)$ quantile of the standard normal distribution.

Construction of funnel plots for the estimated Log-SIR requirse the respective variance of the parameter ($\sigma _j$) estimated either using the Delta-method or re-smapling techniques such as bootstrapping or Bayesian MCMC [1, 29, 30].

Accordingly, since $E_j$ is estimated from a logistic model, accounting for parameter uncertainty, the total variance for center j becomes:

14 $$\begin{aligned} \text {Var}[\log (\text {SIR}_j)] & = \text {Var}[\log (O_j)] + \text {Var}[\log (E_j)] \nonumber \\ & \quad - 2\text {Cov}[\log (O_j), \log (E_j)] \end{aligned}$$

which is estimated using Bayesian MCMC approach [29, 30].

#### False Discovery Rate (FDR) adjusted limits

To control the proportion of false signals among all signals, we use FDR-adjusted limits [31]. In the below, we summarized the steps for constructing the funnel plot. For *m* centers:

5. Calculate two-sided p-values for each hospital:
  15 $$\begin{aligned} p_j = 2 \cdot \Phi \left( -\frac{|\log (\text {SIR}_j)|}{\sigma _j}\right) \end{aligned}$$
  where $\Phi$ is the standard normal CDF.
6. Then, order p-values: $p_{(1)} \le p_{(2)} \le \cdots \le p_{(m)}$
7. For FDR level *q*, find the largest *k* such that:
  16 $$\begin{aligned} p_{(k)} \le \frac{k}{m} \cdot q \end{aligned}$$
8. The FDR-adjusted critical value is:
  17 $$\begin{aligned} z_{\text {FDR}} = \Phi ^{-1}\left( 1 - \frac{p_{(k)}}{2}\right) \end{aligned}$$
9. Finally, the FDR-adjusted limits are:
  18 $$\begin{aligned} \text {FDR Limits} = \pm z_{\text {FDR}} \cdot \sigma _j \end{aligned}$$

The hierarchical logistic regression model was implemented in R using a MH algorithm within Gibbs sampler involving 20,000 iterations, a burn-in of 2,000 iterations and a thinig interval of 1. Three independent chains were run. Convergence of chains were assessed using Gelman-Rubin potential scale reduction factor (PSRF) and effective sample size (ESS). Chain-specific proposal variances were tuned during preliminary pilot runs to target acceptance rates in the range of 20–40%, thereby promoting stable mixing and computational efficiency of the Metropolis–Hastings sampler. For assessing the computational performance of the MH algorithm, we also run the same model using R2jags [32] and Stan [33] R packages. We compared the computational wall clock time taken by the three approaches. We also assessed skrinkage effect using emperical logit, shrunken logit, and other meterics such as the degree of pull towards to the mean, distance moved and % moved and volume category.

Analysis was performed using the R Statistical Programming Language and the associated R packages [34–41].

### Data source

The source of data for our motivating example is the ANZDATA registry [42, 43]. The ANZDATA Registry receives, collates and analyses data from centers providing care for patients receiving long-term dialysis or kidney transplantation in Australia and New Zealand. Data submission is voluntary but complete. For this methodological study, we used the data extract provided by ANZDATA for the Return To Country Study (ANZRREQ-471) [43].

### Ethical clearance

The Return to Country study has been designed and is conducted in accordance with the ethical principles outlined in the World Medical Association’s Declaration of Helsinki. The study is based on a scientifically sound protocol, has undergone independent ethical review, and is conducted with respect for the rights, dignity, privacy, and voluntary informed consent of participants. These principles ensure that risks are minimised and participant welfare is prioritised in line with international ethical standards. Ethics approval has been obtained from the relevant Human Research Ethics Committees in accordance with Australian National Standards, including: Northern Territory HREC (2019-3530); Aboriginal Health & Medical Research Council for NSW (2230/24); Queensland Health HREC (HREC/2023/QCH/99606, Nov v4-1732); South Australia GEMS HREC (2023/HRE00209); and Aboriginal Health Council of South Australia AHREC (04-23-1078).

ANZDATA collects real-time data on incident patients commencing renal replacement therapy, changes in treatment modality or treating centre, deaths, transplantations, and other major clinical events such as peritonitis or new cancer diagnoses. In addition, an annual survey is completed by all dialysis and transplant units on all patients with end-stage kidney disease receiving kidney replacement therapy. The ANZDATA Registry is the data custodian and operates under specific ethics, privacy, data collection, and compliance policies, which are actively monitored and adhered to (attached). For the Return to Country study, access to ANZDATA data is obtained through the ANZDATA data request procedure. The study uses de-identified, unit-level data. Re-identification of data does not occur unless separately approved through appropriate ethics and governance processes. Trial registration number ACTRN12623001241628; https://www.anzctr.org.au/Trial/Registration/TrialReview.aspx?id=379101&isReview=true.

This study is funded by the National Health and Medical Research Council of Australia (GNT1158075).

## Results

Data was extracted from 34 centers, labelled alphabetically and sub-divided by Indigenous status (17 Indigenous and 17 Non-Indigenous “centers”), each with a varying number of patients and predictor values. The hybrid Gibbs-MH algorithm for sampling from the full conditionals was run for 20,000 iterations iwith 2,000 burn-in and a thinning interval of 1 for each chain. As a result, a total of 54,000 samples were retained from three chains for obtaining posterior summaries for the parameters of the hiearchical logistic regression model and hence the derived Log-SIR for each center.

### Posterior

#### Posterior summaries of fixed effects

Posterior summaries indicate statistically significant effects for age groups, gender, age, Indigenous status, lung disease, diabetes, obesity, cardiovascular diseases (CVD), referral status, remoteness and time period (Table 1). Accordingly, male patients had a 15% higher chance of receiving transplant at two years since starting kidney replacement therapy (KRT) than female patients. As the age of ESKD patients increased, the odds of being transplanted at two years since the start of KRT decreased by a range of 23%-99%. That is, the age groups $>= 36-46,>= 46-56, >= 56-66$, and $>= 66-76$, respectively, were at 23%, 46%, 68%, 90% and 99% lower odds of receiving transplant at two years following initiation of KRT. Non-Indigenous patients were more than 5 times more likely to have been transplanted within two years since starting KRT compared to Indigenous patients (Table 1). Patients with Lung diseases, and Diabetes, respectively, had 55% and 66% lower odds of receiving transplant within two years. Similarly, Patients with Obesity and later referal status, respectively, had 41% and 60% lower odds of receiving transplant within two years. However, patients with CVD were at 2.8 times higher odds of receving transplant within two years. Looking at MMM scale for remotness, pateints from Regional centers (MMM 2) had 22% higher chance of receiving transplant within two years compared to those from MMM 1 (Metropolitan areas). On the contrary, patients from remote areas (MMM 6) had up to 23% lower odds of receiving transplant within two years. Most recent years indicated significant drops in the odds of receiving kidney transplant. For instance, the time periods 2019-2023, 2015-2018 and 2010-2014, respectively, had 72%, 25% and 21% lower odds of receiving transplant within two years compared to the time period 2006-2009.

**Table 1:** Posterior summaries for fixed effects ($\boldsymbol{\beta }$)

Parameter	Mean	SD	2.5%	97.5%	Odds Ratio (OR)	Lower CrI	Upper CrI
Intercept	-2.38	0.49	-3.49	-1.48	0.09	0.03	0.23
Male	0.14	0.05	0.03	0.24	1.15	1.03	1.28
Age group$\ge$26-36	-0.14	0.13	-0.41	0.11	0.87	0.67	1.12
Age group$\ge$36-46	-0.31	0.12	-0.55	-0.07	0.73	0.57	0.93
Age group$\ge$46-56	-0.82	0.12	-1.06	-0.58	0.44	0.35	0.56
Age group$\ge$56-66	-1.14	0.12	-1.38	-0.91	0.32	0.25	0.40
Age group$\ge$66-76	-2.35	0.13	-2.61	-2.09	0.10	0.07	0.12
Age group 76+	-5.25	0.36	-6.02	-4.59	0.01	0.00	0.01
Non_Indigenous	1.73	0.24	1.27	2.20	5.63	3.57	9.00
Lung disease	-0.81	0.12	-1.05	-0.57	0.45	0.35	0.56
Diabetes	-1.08	0.06	-1.20	-0.95	0.34	0.30	0.39
CVD	1.04	0.39	0.37	1.99	2.84	1.44	7.33
Late referral	-0.93	0.08	-1.09	-0.76	0.40	0.33	0.47
Obesity	-0.52	0.06	-0.64	-0.40	0.59	0.53	0.67
MMM 2	0.20	0.10	0.00	0.40	1.22	1.00	1.49
MMM 3	-0.02	0.13	-0.27	0.24	0.98	0.76	1.27
MMM 4	-0.02	0.16	-0.33	0.28	0.98	0.72	1.32
MMM 5	0.08	0.09	-0.09	0.25	1.08	0.91	1.28
MMM 6	-0.31	0.16	-0.63	0.00	0.73	0.53	1.00
MMM 7	0.11	0.17	-0.22	0.43	1.12	0.80	1.54
Time period 2010-2014	-0.23	0.07	-0.38	-0.09	0.79	0.69	0.92
Time period 2015-2018	-0.29	0.08	-0.44	-0.14	0.75	0.64	0.87
Time period 2019-2023	-1.27	0.08	-1.44	-1.11	0.28	0.24	0.33

The parameter estimates in this table are from 20,000 iterations with 2,000 burn-in and a thinning interval of 1, and 3 chains

https://www.health.gov.au/topics/rural-health-workforce/classifications/mmm?language=en

*CrI* Credible Interval, *MMM* Modified Monash Model (MMM) for remoteness defines whether a location is metropolitan, rural, remote or very remote

#### Posterior summaries of random effects ($u_j$)

There was substantial between-centre variability, with certain centers demonstrating notably higher or lower transplant odds compared to the average (Table 2).

**Table 2:** Posterior summaries for centre-specific random intercepts ($u_j$)

Centre	Mean	SD	2.5%	97.5%	Odds Ratio	Lower CrI	Upper CrI
AI	-0.12	0.44	-1.06	0.70	0.89	0.35	2.01
AN	-0.25	0.29	-0.80	0.30	0.78	0.45	1.35
BI	-0.82	0.33	-1.45	-0.16	0.44	0.23	0.85
BN	-0.32	0.24	-0.75	0.16	0.73	0.47	1.17
CI	-0.33	0.31	-0.92	0.29	0.72	0.40	1.34
CN	-0.22	0.20	-0.59	0.19	0.80	0.55	1.21
DI	0.68	0.32	0.04	1.25	1.97	1.04	3.49
DN	0.71	0.15	0.45	1.02	2.03	1.57	2.77
EI	-0.33	0.35	-1.07	0.31	0.72	0.34	1.36
EN	-0.48	0.22	-0.90	-0.04	0.62	0.41	0.96
FI	-0.32	0.52	-1.54	0.54	0.73	0.21	1.72
FN	-0.39	0.16	-0.67	-0.07	0.68	0.51	0.93
GI	-0.40	0.52	-1.55	0.50	0.67	0.21	1.65
GN	-0.13	0.23	-0.58	0.31	0.88	0.56	1.36
HI	0.08	0.46	-0.77	1.00	1.08	0.46	2.72
HN	0.03	0.25	-0.44	0.50	1.03	0.64	1.65
II	-0.38	0.46	-1.32	0.49	0.68	0.27	1.63
IN	-0.05	0.16	-0.34	0.27	0.95	0.71	1.31
JI	0.36	0.38	-0.40	1.06	1.43	0.67	2.89
JN	0.66	0.16	0.37	0.98	1.93	1.45	2.66
OI	-0.03	0.44	-0.86	0.85	0.97	0.42	2.34
ON	0.03	0.16	-0.26	0.34	1.03	0.77	1.40
PI	-0.23	0.54	-1.29	0.89	0.79	0.28	2.44
PN	-0.15	0.24	-0.62	0.33	0.86	0.54	1.39
QI	-0.57	0.35	-1.30	0.05	0.57	0.27	1.05
QN	-0.05	0.47	-0.97	0.88	0.95	0.38	2.41
RI	-0.16	0.46	-1.11	0.67	0.85	0.33	1.95
RN	-0.26	0.24	-0.73	0.21	0.77	0.48	1.23
SI	0.60	0.41	-0.14	1.39	1.82	0.87	4.01
SN	-0.12	0.17	-0.44	0.24	0.89	0.64	1.27
TI	0.72	0.38	-0.11	1.41	2.05	0.90	4.10
TN	0.77	0.18	0.44	1.14	2.16	1.55	3.13
UI	0.26	0.41	-0.70	1.00	1.30	0.50	2.72
UN	0.08	0.19	-0.29	0.46	1.08	0.75	1.58

#### Posterior summaries of Log-SIRs

**Fig. 1:**
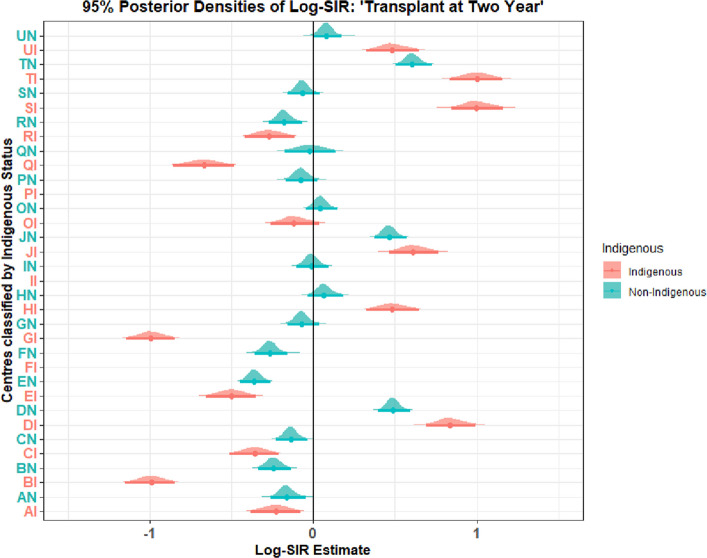
Forest plot of posterior Log-SIR assess centers’ performance in providing kidney transplant at two years for patients starting KRT presenting 95% posterior distribution

Table 3 provides a rich summary of center performance, framed in terms of the log Standardized Incidence Ratio (Log-SIR). The key is to understand that a Log-SIR of 0.0 means the center’s incidence is exactly as expected. A Log-SIR significantly above 0.0 indicates higher-than-expected incidence, and a Log-SIR significantly below 0.0 indicates lower-than-expected incidence. The predicted number of ESKD patients receiving transplant per centre showed strong agreement with empirical kidney transplant patterns. Accordingly, the posterior summaries of the Log-SIR for the centers analyzed revealed substantial variation in performance, with several centers demonstrating incidence rates statistically divergent from the expected value. The 95% posterior credible intervals (CrI) identified clear performance outliers. Nine centers exhibited significantly higher-than-expected incidence, with their entire CrI above zero on the log-scale (equivalent to an SIR $> 1$): DI (Log-SIR = 0.84, 95% CrI: 0.53 - 1.16 ) and DN (Log-SIR = 0.49, 95% CrI: 0.34 - 0.63), HI (Log-SIR = 0.49, 95% CrI: 0.16 - 0.82), HN (Log-SIR = 0.07, 95% CrI: $-0.10$ - 0.24), JI (Log-SIR = 0.61, 95% CrI: 0.03 - 0.94), and JN (Log-SIR = 0.46, 95% CrI: 0.31- 0.61), SI (Log-SIR = 1.00, 95% CrI: 0.68 - 1.33), TI (Log-SIR = 1.00, 95% CrI: 0.67 - 1.34) and TN (Log-SIR = 0.61, 95% CrI: 0.44 - 0.77), and UN (Log-SIR = 0.08, 95% CrI: -0.06 - 0.22 ) (Table 3 and Fig. 1).

**Table 3:** Posterior summaries for centre-specific Log-SIRs

Centre	Log-SIR	SD	2.5%	97.5%	SIR	Lower CrI	Upper CrI
AI	-0.23	0.17	-0.55	0.10	0.80	0.58	1.11
AN	-0.16	0.09	-0.32	0.01	0.85	0.72	1.01
BI	-0.99	0.16	-1.30	-0.67	0.37	0.27	0.51
BN	-0.24	0.08	-0.40	-0.07	0.79	0.67	0.93
CI	-0.36	0.16	-0.67	-0.04	0.70	0.51	0.96
CN	-0.14	0.08	-0.29	0.01	0.87	0.75	1.01
DI	0.84	0.16	0.53	1.16	2.31	1.70	3.18
DN	0.49	0.08	0.34	0.63	1.63	1.41	1.89
EI	-0.50	0.16	-0.82	-0.18	0.61	0.44	0.84
EN	-0.36	0.08	-0.51	-0.21	0.70	0.60	0.81
FI	-14.21	0.17	-14.54	-13.88	0.00	0.00	0.00
FN	-0.26	0.08	-0.42	-0.11	0.77	0.66	0.89
GI	-0.99	0.16	-1.30	-0.67	0.37	0.27	0.51
GN	-0.07	0.08	-0.22	0.08	0.93	0.80	1.09
HI	0.49	0.17	0.16	0.82	1.63	1.17	2.27
HN	0.07	0.09	-0.10	0.24	1.07	0.91	1.28
II	-14.40	0.16	-14.71	-14.08	0.00	0.00	0.00
IN	-0.01	0.08	-0.16	0.14	0.99	0.85	1.15
JI	0.61	0.16	0.30	0.94	1.85	1.35	2.56
JN	0.46	0.08	0.31	0.61	1.59	1.37	1.85
OI	-0.11	0.16	-0.42	0.20	0.89	0.66	1.22
ON	0.05	0.08	-0.10	0.19	1.05	0.90	1.21
PI	-13.50	0.17	-13.82	-13.17	0.00	0.00	0.00
PN	-0.07	0.08	-0.23	0.08	0.93	0.79	1.09
QI	-0.67	0.18	-1.03	-0.30	0.51	0.36	0.74
QN	-0.02	0.14	-0.28	0.25	0.98	0.75	1.28
RI	-0.27	0.16	-0.58	0.06	0.77	0.56	1.06
RN	-0.18	0.08	-0.33	-0.01	0.84	0.72	0.99
SI	1.00	0.17	0.68	1.33	2.72	1.98	3.77
SN	-0.07	0.08	-0.21	0.08	0.94	0.81	1.09
TI	1.00	0.17	0.67	1.34	2.72	1.96	3.82
TN	0.61	0.09	0.44	0.77	1.84	1.55	2.17
UI	0.49	0.17	0.17	0.81	1.63	1.18	2.25
UN	0.08	0.07	-0.06	0.22	1.08	0.94	1.25

Conversely, thirteen centers exhibited significantly lower-than-expected incidence, with CrI entirely below zero (equivalent to an SIR $$< 1$$): BI (Log-SIR = $$-0.99$$, 95% CrI: $$-1.30$$ - $$-.67$$), BN (Log-SIR = $$-0.24$$, 95% CrI: $$-0.40$$ - $$-0.07$$), EI (Log-SIR = $$-0.50$$, 95% CrI: $$-0.82$$ - $$-018$$), EN (Log-SIR = $$-0.36$$, 95% CrI: $$-0.51$$ - $$-0.21$$), FN (Log-SIR = $$-0.26$$, 95% CrI: $$-0.42$$ - $$-0.11$$), GI (Log-SIR = $$-0.99$$, 95% CrI: $$-1.30$$ - $$-0.67$$), QI (Log-SIR = $$-0.67$$, 95% CrI: $$ -1.03$$ - $$-.30$$), RN (Log-SIR = $$-0.18$$, 95% CrI: $$-0.338$$ - $$-0.01$$).

Two centers represented by FI and II had zero counts of transplanted patients within two years and their Log-SIR were undefined. To aviod this numerical issues during computation, we have used $1e-6$ term both in numerator and denomunator. Hence, the resulting Log-SIR for the centers were respectively, FI (Log-SIR = $-14.21$, 95% CrI: $-14.54$ - $-13.88$), II (Log-SIR = $-14.40$, 95% CrI: $-14.71$ - $-14.08$). Center PI had the lowest Log-SIR (Log-SIR = $-13.50$, 95% CrI: $-13.82$ - $-13.17$) (Fig. 3).

A notable pattern was observed between centre designations. The “I” centers comprised all high-incidence outliers and three of the four low-incidence outliers, indicating more extreme performance outcomes. In contrast, the posterior credible intervals for nearly all “N” centers contained zero (Log-SIR $\approx$ 0, equivalent to SIR $\approx$ 1), suggesting their incidence rates were not statistically distinguishable from the expected rate. This dichotomy points to a potential difference in case-mix complexity, patient risk, or service specialization between the two designations (Fig. 1).

For the remaining majority of centers, the posterior credible intervals for the log-SIR contained zero, indicating the data were insufficient to conclude that their performance was statistically different from the expected benchmark.

**Fig. 2:**
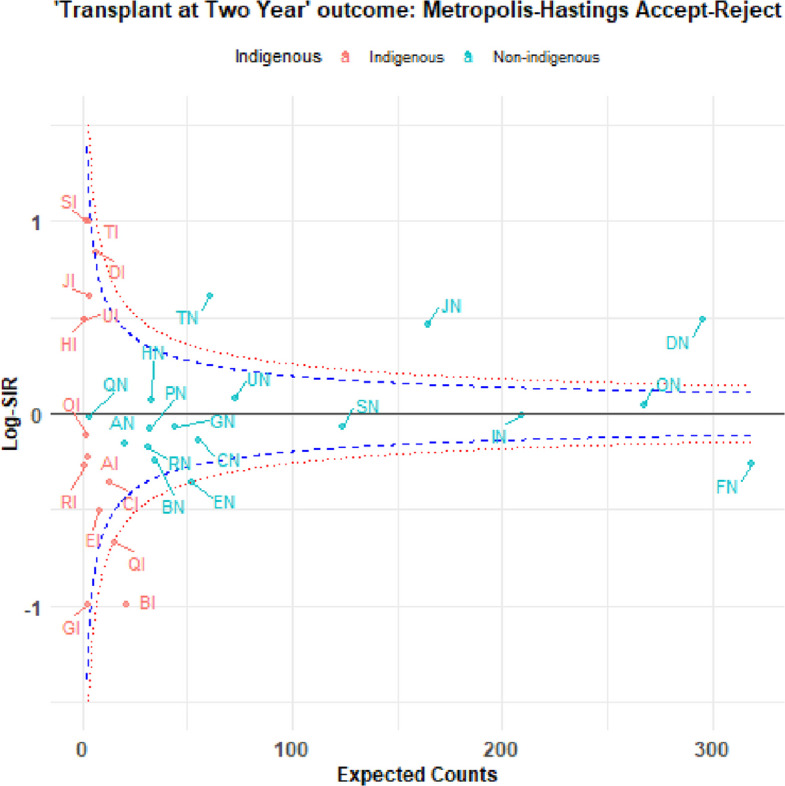
Funnel plot for Log-SIR assess centers’ performance in providing kdiney transplant at two years for patients starting KRT

Figure 2 presents a funnel plot that compares the performance of centers using Log-SIR. centers within the upper and lower False Discovery Rates (FDRs) indicate expected performance in transplanting patients close to home (are in the region of average performance). The dashed lines forming funnels around the horizontal solid line (Log-SIR=0) indicate expected variation, with centers falling outside these limits exhibiting statistically significant differences from the norm.

Overall, larger centers exhibit more stable Log-SIR values, while smaller centers experience greater variation, reinforcing the importance of centre size in the assessment of centers’ performance in provision of kidney transplant within two years starting KRT. Using this method, the variance of Log-SIR appears relatively low, with most values concentrated around zero. Some extreme values (outliers) are present on the left-hand side, indicating a few centers with more deviation.

These findings reinforce the usefulness of hierarchical Bayesian models in stabilizing kidney transplant rate estimates and hence enhancing comparabilty of centers, especially for centers with limited patient volume. This probabilistic framework supports transparent and evidence-based decision-making by allowing stakeholders to set explicit risk tolerances (e.g., classifying a provider as an outlier if there is a 95% probability their effect exceeds a benchmark). Our study employed posterior tail probabilities to identify providers with significantly higher-than-expected outcome rates, aligning with best practices in Bayesian decision theory.

To this end, the forest plot (Fig. 2) provides a decision-theoretic classification rules via posterior tail probabilities. It visualizes the posterior estimates of the Log-SIR for different centers, with a clear distinction between those serving Indigenoous and Non-Indigenous populations. The central vertical line at a Log-SIR of 0 represents the expected incidence rate. A centre’s credible interval overlapping this line suggests its incidence is not statistically different from the expected rate. Intervals entirely to the right indicate significantly higher-than-expected incidence, while intervals entirely to the left indicate significantly lower-than-expected incidence.

There is a striking and consistent pattern between the two groups. The centers represented in Indigenous predominantly have Log-SIR estimates greater than or less than 0 ($\text {SIR} > 1.0 \ \text {or} \ < 1.0$), with some of their credible intervals lying entirely to the right of the null line and some entirely to the left of the null line. This indicates that observed heterogeneity was more pronounced among Indigenous-serving centers, respectively, higher and lower incidence of the measured outcome compared to the expected rate.

Conversely, eight centers represented in Non-Indigenous were almost exclusively clustered around the null region of the plot. Their Log-SIR estimates with credible intervals crossing the null line. This indicates that, for those Non-Indigenous centers, they had incidence equivalent to expected.

The width of the centre lines (credible intervals) varies, indicating different levels of precision in the estimates for each centre. Centers with shorter lines have more precise estimates (likely due to larger sample sizes), while those with longer lines have more statistical uncertainty.

**Fig. 3:**
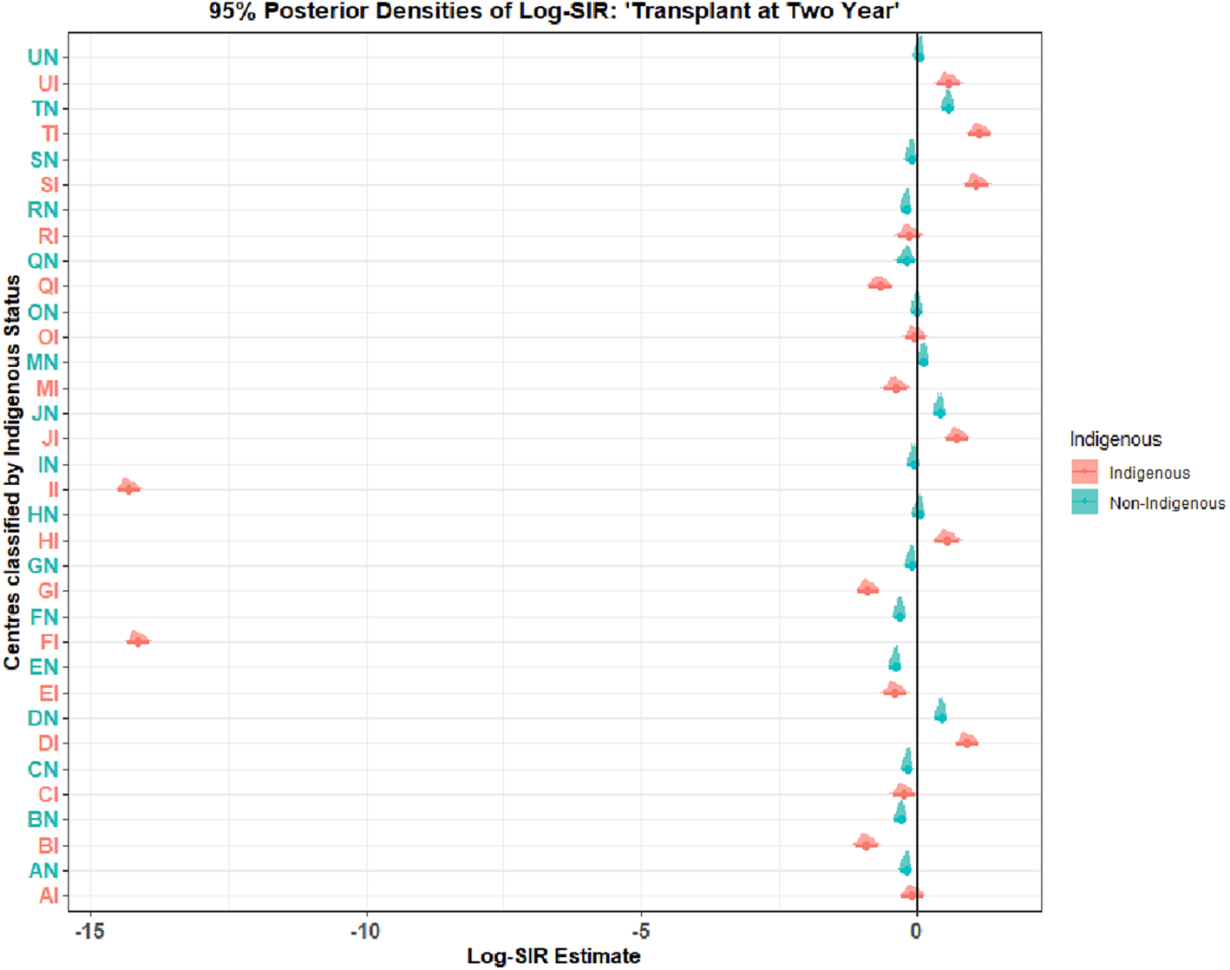
Funnel plot for Log-SIR assess centers’ performance in providing kidney transplant at two years for patients starting KRT. Note: the plot displays all centers including centers with zero kidney transplant status at two years

Close examination of the data, sparsity was a probable issue for numerical instabilit, espcailly for convergence estimates (slightly higher $\text {PSRF} >1.0$). For instance, CVD and Indigenous status had low counts on the outcome variable (Table 4). Also, we observed extremely low derived estimates on the Log-SIR due to zero count on the outcome (center FI and II). Figure 3 presents a forest plot with all centers including those centers with zero counts on kidney transplant within two years.

**Table 4:** Transplanted within two years of starting kidney replacement therapy

Transplanted	No	Yes
CVD Yes	333	4
CVD No	15110	2098
Indigenous	3463	76
Non-Indigenous	11980	2026

### Assessing model convergence

Model convergence was assessed using Gelman-Rubin Potential Scale Reduction Factor (PSRF) and Effective Sample Size (ESS). Table 5 presents Gelman-Rubin Statistic PSRF and ESS for the regression parameters. PSRF values close to 1 (typically $<1.1$) indicate good convergence of MCMC chains [44]. The Gelman-Rubin PSRF shown in Table 5 for the parameters fall between 1.000 and 1.157, suggesting good convergence.

**Table 5:** Gelman-Rubin diagnostic and effective sample size

Parameter	Gelman-Rubin	Effective sample size
Intercept	1.093902	31.97269
Male	1.001238	3449.19089
Age group $\ge$26-36	1.002057	740.17035
Age group $\ge$36-46	1.004413	631.16519
Age group $\ge$46-56	1.004750	596.91841
Age group $\ge$56-66	1.005129	592.01776
Age group $\ge$66-76	1.002966	732.99272
76+	1.000898	1733.45327
Non_Indigenous	1.157055	121.34691
Lung diseases	1.000608	6763.56142
Diabetes	1.000652	6501.27626
CVD	1.083243	50.83549
Late referral	1.002298	7889.84155
Obesity	1.000225	7072.40174
MMM 2	1.000137	2027.05619
MMM 3	1.000970	4006.06900
MMM 4	1.003469	2874.69371
MMM 5	1.000478	2019.83676
MMM 6	1.003340	1947.25709
MMM 7	1.000553	1401.35099
Time period 2010-2014	1.003090	2664.27228
Time period 2015-2018	1.012098	2529.01249
Time period 2019-2023	1.008091	2549.82616

The PSRF for Non-Indigenous predictor was slightly higher than 1.0 (PSRF = 1.157). A PSRF higher than 1.1 can occur for several reasons, such as strong correlations between the intercept and other parameters (e.g., group-level random effects) [44], poor initialization of chains (i.e., if chains start from very different values) [45], weak identifiability (such as sparse data or unbalanced groups, leading to a broad or multi-modal posterior for the intercept) [46], and the choice of centered vs. non-centered parameterization, which significantly affects convergence in hierarchical models [47, 48]. However, our analysis indicated that adding more iterations had significant improvement in reducing the PSRF close to 1.0. For instance, the PSRF for Non-Indigenous predictor was 1.181 (Table 10 in Appendix A) with 10000 iterations and with 20000 iterations become 1.157 (Table 11 in Appendix A).

In addition, the PSRF for CVD was 1.261 (Table 10 in Appendix A) exceeding the 1.1 threshold with 10000 iterations suggesting potential non-convergence; with 20000 iterations, the PSRF become 1.083 (Table 11 in Appendix A). The groupings in the data for CVD categories are imbalanced or sparsely represented across clusters, and hence this can lead to slow convergence [46–48]. The ESS reflects how many independent samples the MCMC has effectively drawn. The ESS values $>200-300$ are usually sufficient for stable posterior estimates [15]. Most parameters here had high ESS (many well over 1000). However, the intercept had 31.97 ESS suggesting very low; unreliable estimate. Similarly, the ESS for CVD, Non-Indigenous, and age group $>=56-66$, respectively, are 50.83 and 121.34 requiring more sampling or better tuning.

Table 6 presents Gelman-Rubin PSRF and ESS for the random effects. In this table, most parameters have values between 1.000 and 1.01, suggesting excellent convergence. However, centers AI (Centre “A”, Indigenous, PSRF = 1.17), FI (Centre “F”, Indigenous, PSRF = 1.16) and GI (Centre “F”, Indigenous, PSRF = 1.14), exceeded the commonly accepted threshold and might warrant further sampling or diagnostics. Most ESS values were below 300, which is not adequate enough suggesting the need for longer chains (Table 6). Overall, convergence is satisfactory for nearly all parameters with minor concerns due to low Gelman-Rubin statistics.

**Table 6:** Gelman-Rubin diagnostic and effective sample size for the centre-level random effects

Parameter(u)	Gelman-rubin	Effective sample size
AI	1.172746	66.11442
AN	1.075918	138.20905
BI	1.015170	107.20143
BN	1.032919	215.02761
CI	1.045911	128.75336
CN	1.077097	283.26193
DI	1.013479	122.06204
DN	1.052178	133.25058
EI	1.074813	89.80851
EN	1.005926	199.25524
FI	1.164921	51.98890
FN	1.045541	173.55801
GI	1.143898	47.34481
GN	1.034620	221.93803
HI	1.153377	59.94620
HN	1.017882	188.96059
II	1.067676	60.47347
IN	1.035479	214.22826
JI	1.002472	89.50210
JN	1.055786	196.36466
OI	1.019000	63.61830
ON	1.055584	168.54977
PI	1.059857	45.00012
PN	1.000907	173.93432
QI	1.039505	100.21944
QN	1.061048	60.80124
RI	1.018069	60.26440
RN	1.082962	200.88686
SI	1.068816	70.89371
SN	1.045102	255.18947
TI	1.075738	83.52723
TN	1.042632	310.78814
UI	1.033237	73.22652
UN	1.042885	269.00218

Trace plots for all three methods exhibit good mixing with no visible trends or chain separation, indicating adequate convergence (Fig. 5 of Appendix A). Posterior density plots show strong overlap between chains within each method, suggesting stable marginal distributions and negligible sensitivity to initial values (Fig. 6 of Appendix A).

### MH, R2jags and Stan comparison

Comparison of analysis outputs from our custom MH, and the standanrd Stan and R2jags R packages are presented in (Tables 8 through 10 in Appendix A). The comparison of the three approaches involved 10,000 iterations, 3 chains and a thining interval of 1.

Across the three Bayesian sampling approaches (custom MH, R2jags, and Stan), posterior summaries including the mean, median, standard deviation, and central credible intervals (2.5%, 25%, 50%, 75%, and 97.5%) were highly consistent, indicating that all samplers approximated the same target posterior distribution. Minor differences were observed in the convergence diagnostics. Both R2jags and Stan produced $\hat{R}$ values very close to 1.0 and relatively large effective sample sizes ($n_{\text {eff}}$), whereas the MH sampler exhibited slightly elevated $\hat{R}$ values (up to approximately 1.15) and smaller $n_{\text {eff}}$ for sparse covariates (for instance, Indigenous status and CVD covariates) and the intercept term. Values of $\hat{R}$ close to unity generally indicate good mixing and convergence across chains, while low effective sample sizes reflect stronger autocorrelation and reduced sampling efficiency [49]. The superior effective sample sizes observed for Stan are consistent with the use of Hamiltonian Monte Carlo and the No-U-Turn Sampler, which reduce random-walk behavior and improve exploration of high-dimensional posterior distributions [50]. In contrast, random-walk Metropolis–Hastings algorithms are known to mix more slowly and to be sensitive to proposal tuning, particularly in sparse or weakly identified parameter settings. Empirical comparisons further show that Stan typically achieves higher sampling efficiency than Gibbs-based implementations such as JAGS, although posterior point estimates remain comparable when convergence is achieved [51]. Overall, the close agreement in posterior summaries supports the robustness of the substantive inference, while the observed differences in $\hat{R}$ and $n_{\text {eff}}$ primarily reflect algorithmic efficiency rather than systematic discrepancies in parameter estimation.

We have provided an expanded discussion of the findings in light of computational efficiency of HM, R2jags and Stan in the Discussion section comparing the number of iterations required and convergence using PSRF and ESS (Tables 8 through 10 in Appendix A) and the wall-clock time taken (Table 12 in Appendix A).

### Posterior predictive check

**Fig. 4:**
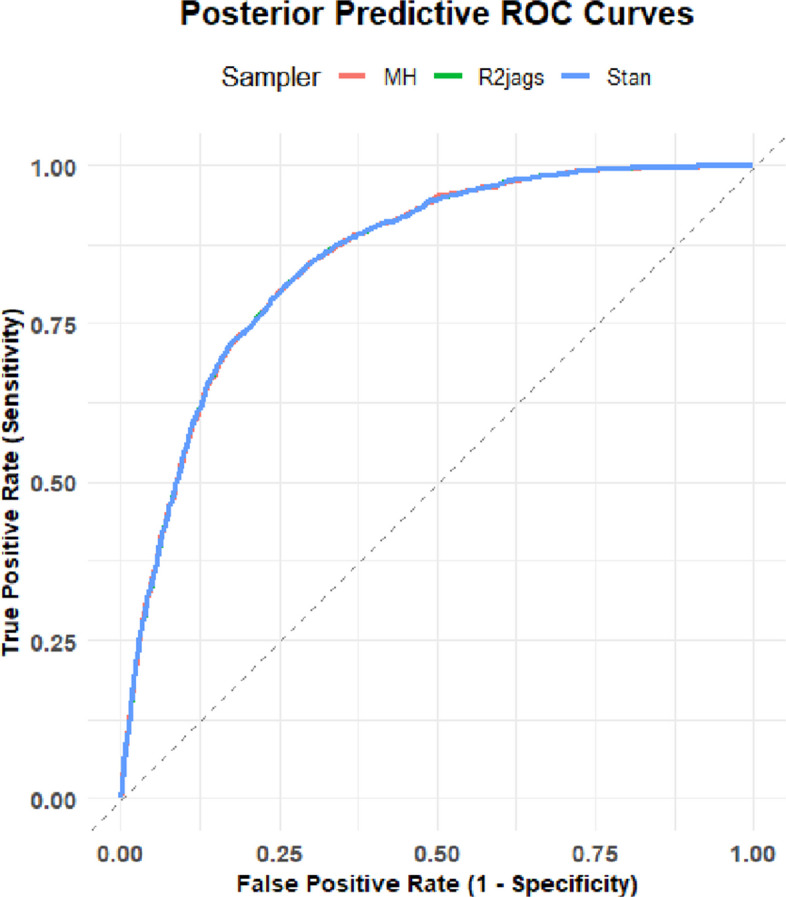
ROC Curves assess predictive performance of MH, R2jags and Stan

**Fig. 5:**
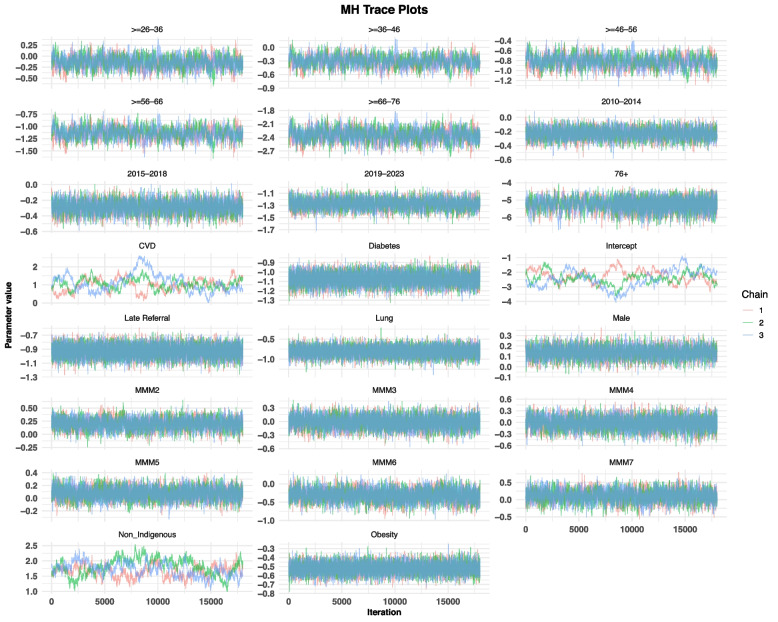
Posterior density plots of regression coefficients obtained from the Metropolis–Hastings sampler

**Fig. 6:**
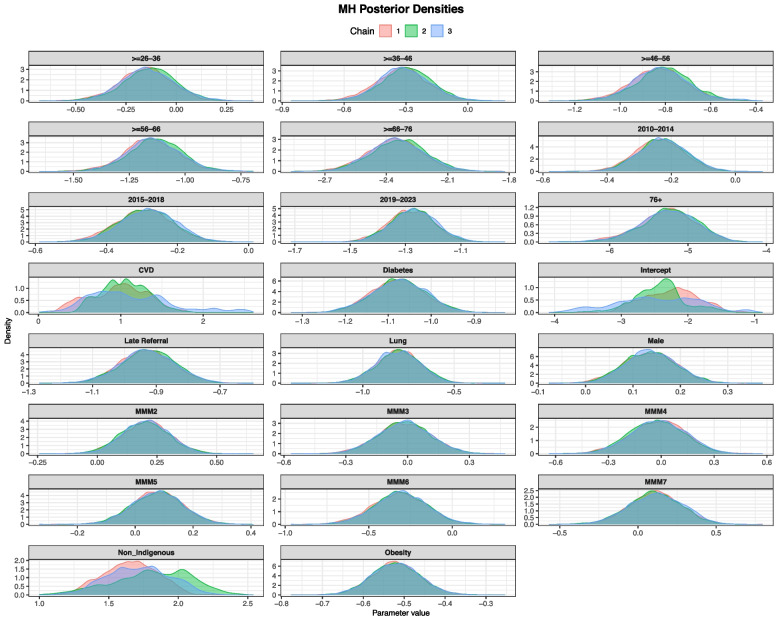
Posterior density plots of regression coefficients obtained from the Metropolis–Hastings sampler

Posterior predictive performance was evaluated using receiver operating characteristic (ROC) curves and the area under the curve (AUC). Posterior predictive probabilities were obtained by averaging predicted probabilities across posterior draws for each sampler. All three methods yielded nearly identical ROC curves and overlapping posterior distributions of AUC, indicating comparable predictive performance (85%) and reinforcing the equivalence of posterior inference across sampling algorithms (Table 7 and Fig. 4).

**Table 7:** Quantiles of AUC draws for different models (values rounded to four decimal places)

Method	2.5%	50%	97.5%
MH	84.90	85.12	85.23
R2jags	84.92	85.13	85.23
Stan	84.91	85.13	85.23

It is evideent that the posterior quantiles of AUC were nearly identical across the MH, R2jags, and Stan implementations, with substantial overlap of the corresponding posterior intervals. In addition, the overlaid ROC curves were visually indistinguishable, indicating consistent discrimination performance and identical ranking behavior across all classification thresholds. These results demonstrate that predictive inference is robust to the choice of sampling algorithm and that any remaining differences among samplers primarily reflect computational efficiency rather than substantive differences in predictive accuracy or uncertainty.

## Discussion

Bayesian hierarchical logistic regression (BHLR) provides a robust statistical framework for evaluating hospital and provider performance, particularly in settings where patient outcomes are influenced by both individual characteristics and provider-level factors [10, 12, 13].

We emphasise that, regardless of the estimation strategy, Bayesian hierarchical models offer significant advantages for provider profiling, including: adjustment for patient-level risk (case-mix), ensuring fair comparisons across providers with different patient populations [12], shrinkage of hospital-level effects, preventing overinterpretation of random variability [13] and inclusion of low-volume providers, enhancing the comprehensiveness and comparability of performance assessments [10] and decision-theoretic classification via posterior tail probabilities, allowing for principled identification of outlier providers [14].

R2jags provides a user-friendly interface to JAGS, a flexible MCMC engine based on Gibbs sampling. JAGS automatically incorporates adaptive Metropolis-Hastings updates where required for non-conjugate models, streamlining model fitting for practitioners [10]. The strength of using JAGS lies in its model-building flexibility and reduced programming burden, allowing for rapid prototyping and robust diagnostics. However, manual control of the sampling algorithm, as provided by Gibbs-MH routines, can offer advantages in fine-tuning and understanding the behavior of the sampling process, especially for complex hierarchical models or when exploring alternative priors [10].

In hierarchical logistic models, where the logistic link leads to non-conjugate posterior distributions, the Metropolis-Hastings algorithm facilitates efficient sampling from full conditionals, ensuring convergence and adequate posterior exploration [13, 14].

Therefore, this study presents findings of Gibbs sampling with Metropolis-Hastings (MH) steps producing consistent results in terms of parameter estimates, posterior distributions, and provider classifications similar to our previous work implemented using R2jags [30]. This implementation aligns with the approach advocated by Austin (2008), who highlighted the importance of Bayesian hierarchical modeling in provider profiling, emphasizing control over shrinkage effects and posterior inference [13]. Similarly, Austin & Brunner (2007) leveraged Bayesian posterior tail probabilities in profiling high-mortality hospitals, demonstrating the practical impact of well-calibrated MCMC estimation [14].

In our implementation, Gibbs sampling cycles were constructed where the random effects were updated using Gibbs steps when possible, and the fixed effects and variance components were updated using Metropolis-Hastings proposals due to the logistic likelihood’s non-conjugate form [15].

Manual implementation of Gibbs sampling with embedded Metropolis-Hastings updates offers researchers fine-grained control over the sampling process, including proposal distributions, tuning parameters, and acceptance rates. This approach is particularly useful for methodological research where custom modeling or experimentation with priors and update rules is required [13, 14].

The use of MH steps allowed precise control over proposal distributions and tuning parameters, which proved effective in achieving good mixing and convergence across chains. However, random-walk MH and Gibbs samplers are known to be simple and widely applicable but can suffer from poor mixing and low ESS in hierarchical or high-dimensional models due to local move limitations [15, 16].

This implementation achieved stable convergence across chains, with effective sample sizes comparable to those generated by automated methods except the covariates with sparse data. This highlights the viability of Metropolis-within-Gibbs algorithms for hierarchical healthcare performance assessment, especially when tailored control over the estimation process is desired. Our empirical observations align closely with broader findings in the Bayesian computation literature. For instance, R2jags is particularly advantageous for applied research contexts where model flexibility and rapid iteration are important, offering built-in convergence diagnostics and user-friendly syntax.

NUTS/HMC shows superior performance in models with complex posterior geometry because the use of derivatives allows proposals that traverse posterior contours more effectively than uninformed proposals [19, 20].

Comparisons between Stan and other MCMC engines consistently show that, while Stan may require more computation per iteration, the quality and efficiency of samples often justify the additional time, especially when accurate inference is critical [52, 53].

Thus, while our MH and R2jags runs were faster in raw time, the effective performance per unit of ESS likely favors Stan, a conclusion that has been echoed in simulation studies and applied comparisons in the literature [49, 54].

Practical implications for applied modeling, our comparison highlights a practical trade-off. MH and R2jags can be competitive in wall-clock time and are often easier to implement for simpler models or exploratory analysis. Stan’s HMC/NUTS demands more compute time upfront but generally yields better mixing, more reliable diagnostics, and higher effective sample sizes, which are particularly valuable in hierarchical models with non-Gaussian posteriors or sparse data.

Moreover, shrinkage analysis reveals clear patterns consistent with well-documented phenomena in hierarchical modelling, particularly the stabilising effect of partial pooling in the presence of differing sample sizes (centers with low versus high volume) (Table 13 in Appendix A).

Accordingly, across centers, shrinkage tends to pull the empirical logit estimates toward the group mean, with the direction and magnitude of adjustment driven largely by the degree of uncertainty around each center’s empirical estimate. This pattern reflects the classic behaviour of hierarchical models in the presence of sparse data: estimates from centers with fewer observations or rare events are “shrunk” more toward the overall mean to mitigate over-interpretation of noisy rates [55, 56].

In contrast, centers with large patient volume and more stable empirical logits often exhibit smaller adjustments. Some large centers even shift slightly away from the mean reflecting the data’s strength in overriding the hierarchical prior to some extent. This is consistent with the principle that higher-volume groups yield more precise within-group estimates that require less shrinkage [57]. This volume-shrinkage relationship is a fundamental feature of hierarchical modelling: as sample size increases, the posterior for group effects is increasingly dominated by the group’s data rather than the group mean prior [58, 59].

These findings manifest well-established results in hierarchical and/or small-area estimation. The shrinkage phenomenon observed aligns with the seminal work of Efron and Morris (1975), who demonstrated that in the presence of many groups with varying sample sizes, hierarchical models that perform partial pooling often yield estimates with lower overall mean squared error compared to separate (non-hierarchical) estimates [59]. Gelman et al. (2006) and Raudenbush and Bryk (2002) further articulate how hierarchical Bayesian models adaptively balance within-group information and between-group variance, particularly when group sample sizes differ substantially [55, 58].

More recent applications in healthcare quality assessment reflect similar shrinkage patterns, where low-volume providers’ performance metrics are “shrunk” more strongly toward the overall mean to avoid over-interpretation of random noise [60, 61]. Such adjustments are particularly critical in binary outcome models, where rare events (e.g., adverse events) can lead to extreme empirical logits that do not reflect reliable signals without shrinkage.

Practically, this shrinkage analysis suggests that care should be taken when interpreting raw empirical performance metrics for centers with small or moderate patient volumes. Without partial pooling, extreme empirical logits at low-volume centers may be misinterpreted as true effects rather than sampling variation. The hierarchical shrinkage approach stabilises these estimates, producing more robust and conservative metrics that better reflect the true underlying center performance.

This pattern supports policy and clinical decision frameworks that favour hierarchical or empirical Bayes adjustments in performance reporting, as discussed in health services research [60, 61].

For an expanded discussion and detailed results of comptational time and shrinkage effect on the parameters of the model, refer to Appendix A “Computational time” and “Shrinkage”, respectively.

This study has several limitations. First, the logistic likelihood is non-conjugate, requiring careful proposal tuning; poor tuning can lead to slow mixing or biased estimates. Second, posterior inferences can be sensitive to parameter initialization, prior specification, and model parameterization (e.g., centered vs. non-centered), which may affect both parameter estimates and derived quantities such as predicted probabilities and the Log-SIR. Thirdly, SIR results derived from non-converged chains for some of the parameters are and interpretation of the findings should be with caution especially for those centres with small volumes and sparse data for some of the covariates. Finally, numerical instability due to potential separation in sparse data (as in the case of CVD), and the computational burden of scaling to complex hierarchical structures may further limit efficiency and robustness.

## Conclusions

In summary, these results support the conclusion that the custom Metropolis–Hastings implementation provides valid and reliable posterior inference for the present application despite the reduced computational efficiency for certain parameters relative to R2jags and Stan. The observed convergence behavior does not materially affect the substantive interpretation of the model outputs, but it highlights the practical advantages of modern sampling algorithms when computational efficiency or scalability is a primary concern. From an applied perspective, the consistency across methods strengthens confidence in the reported findings and underscores the methodological robustness of the analysis. Our manual Gibbs sampling with Metropolis-Hastings resulted in robust posterior estimates of fixed effects and random effects, consistent identification of providers with extreme outcomes, as determined by posterior tail probabilities and comparable convergence diagnostics and mixing behavior, underscoring the reliability of our approach for applied health services research. The approach is a counter part of the Bayesian framework that we have implemented using R2jags for assessing centers’/hospitals’ performance in the provision of equitable health services for KRT in Australia [30]. The method is flexible for complex structured data and allows full probabilistic inference. Application to centre kidney transplant prediction illustrated the model’s utility in health outcomes research.

Future work includes extending the model to Polya-Gamma augmentation of the full conditionals for the parameters of hierarchical logistic regression [62]; a conjugate full conditional and hence replace the MH accept-reject approach and directly sample from the actual distributions via Gibbs sampling.

## Supplementary Information

Supplementary Material 1. Additional supporting information may be found in the online version of the article at the publisher’s website.

## Data Availability

Due to confidentiality, we could not share the data used in this research.

## Appendix A: Computational efficienty of MH, R Stan and R2jags

With the purpose of apprasing the computational effeciency of our custom MH algorithm, we implemented three Bayesian estimation strategies for a hierarchical logistic regression model: a custom Metropolis–Hastings (MH) sampler, R2jags as an interface to JAGS’ Gibbs/MH samplers, and Stan using the no-U-turn sampler (NUTS), a variant of Hamiltonian Monte Carlo (HMC). Although all three methods estimate the same model, they differ substantially in computational performance, sampling efficiency, and algorithmic behavior, particularly in the presence of hierarchical structure and potentially sparse data. The comparison of the three approaches involved 10,000 iterations, 3 chains and a thining interval of 1.

### Computational time

In our empirical application, the MH algorithm and R2jags both completed in approximately 54 minutes on the target hardware, whereas the Stan model required roughly 2.84 $\times$ more time (approximately 153.46 minutes) - Table 12. The raw time per iteration for Stan is typically higher due to: Gradient calculations at each leapfrog step; Adaptive tuning of step size and mass matrix; Deep tree exploration during the warmup phase in NUTS [52–54].

These overheads are an inherent part of HMC but often pay dividends in terms of sampling efficiency (see below).

Despite taking longer computational time, Stan’s gradient-based exploration produces lower autocorrelation and generally higher effective sample size (ESS) per iteration compared to MH and R2jags (Tables 8, 9 and 10). In other words, each Stan draw tends to be more statistically informative, reducing the need for extremely long chains compared with MH or R2jags [63]. This trade-off involving higher computation per iteration but greater exploration efficiency is a defining characteristic of HMC compared to random-walk methods.

The performance of MCMC algorithms is not solely a function of runtime. Diagnostics such as the Gelman–Rubin statistic ($\hat{R}$) and effective sample size (ESS) reveal deeper differences:

The MH sampler, while able to produce approximate posterior samples, often shows slow mixing and high autocorrelation in hierarchical structures, especially for group-level variance parameters or when data are sparse within groups. R2jags, depending on the mixing properties of its underlying samplers, may mitigate some of this behavior but still suffers from local random-walk tendencies. Stan’s NUTS typically produces lower $\hat{R}$ values and higher ESS for the same run lengths, indicating more reliable convergence and exploration of the posterior distribution.

These differences are pronounced in hierarchical logistic models, where the posterior often exhibits funnel-like geometry and strong parameter correlations. Gradient-based samplers like HMC are particularly well-suited to navigating such landscapes, which is one reason Stan has become the default engine for many complex Bayesian models [15, 47].

Posterior summaries from Stan model output (the Meteropolis-Hastings Acccept-Reject steps counterpart) is given Table 8 below.

**Table 8:** Stan posterior summary statistics for coefficients of a Hierarchical Logistic Regression Model: 3 chains, each with iter=10000; warmup=2000; thin=5; post-warmup draws per chain=1600, total post-warmup draws=4800

Parameter	Mean	SD	2.5%	25%	50%	75%	97.5%	$\boldsymbol{n}_{\textbf{eff}}$	$\hat{\boldsymbol{R}}$
$\beta [1]$	-2.39	0.39	-3.18	-2.65	-2.39	-2.12	-1.64	19529	1.00
$\beta [2]$	0.11	0.05	0.01	0.08	0.11	0.15	0.22	43590	1.00
$\beta [3]$	-0.17	0.13	-0.43	-0.26	-0.17	-0.09	0.08	12233	1.00
$\beta [4]$	-0.38	0.12	-0.62	-0.46	-0.38	-0.30	-0.14	11600	1.00
$\beta [5]$	-0.84	0.12	-1.07	-0.92	-0.84	-0.76	-0.61	10784	1.00
$\beta [6]$	-1.16	0.12	-1.39	-1.24	-1.16	-1.08	-0.93	10816	1.00
$\beta [7]$	-2.40	0.13	-2.67	-2.49	-2.40	-2.31	-2.15	12253	1.00
$\beta [8]$	-5.35	0.35	-6.07	-5.57	-5.33	-5.11	-4.71	28694	1.00
$\beta [9]$	1.75	0.22	1.31	1.60	1.75	1.89	2.16	7271	1.00
$\beta [10]$	-0.76	0.12	-1.00	-0.84	-0.76	-0.68	-0.54	43846	1.00
$\beta [11]$	-1.08	0.06	-1.20	-1.12	-1.08	-1.04	-0.96	38096	1.00
$\beta [12]$	1.11	0.35	0.46	0.86	1.09	1.33	1.82	25441	1.00
$\beta [13]$	-0.95	0.08	-1.11	-1.00	-0.95	-0.89	-0.79	43318	1.00
$\beta [14]$	-0.52	0.06	-0.64	-0.56	-0.52	-0.49	-0.41	38246	1.00
$\beta [15]$	0.18	0.10	-0.01	0.11	0.18	0.25	0.37	27919	1.00
$\beta [16]$	-0.07	0.13	-0.32	-0.15	-0.07	0.01	0.18	35959	1.00
$\beta [17]$	-0.08	0.16	-0.39	-0.19	-0.08	0.03	0.22	33346	1.00
$\beta [18]$	0.04	0.09	-0.13	-0.02	0.04	0.09	0.21	30917	1.00
$\beta [19]$	-0.14	0.15	-0.44	-0.24	-0.14	-0.04	0.15	30097	1.00
$\beta [20]$	-0.06	0.17	-0.39	-0.17	-0.06	0.05	0.27	31439	1.00
$\beta [21]$	-0.24	0.07	-0.39	-0.29	-0.24	-0.20	-0.10	24413	1.00
$\beta [22]$	-0.31	0.08	-0.46	-0.36	-0.31	-0.26	-0.16	23717	1.00
$\beta [23]$	-1.27	0.08	-1.43	-1.33	-1.27	-1.22	-1.12	24291	1.00

$\beta [1]$ = Intercept, $\beta [2]$ = Male, $\beta [3]$ = Age group $\ge$26-36, $\beta [4]$ = Age group $\ge$36-46, $\beta [5]$ = Age group $\ge$46-56, $\beta [6]$ = Age group $\ge$56-66, $\beta [7]$ = Age group $\ge$66-76, $\beta [8]$ = Age group 76+, $\beta [9]$ = Non-Indigenous, $\beta [10]$ = Lung diseases, $\beta [11]$ = Diabetes, $\beta [12]$ = CVD, $\beta [13]$ = Late referral, $\beta [14]$ = Obesity, $\beta [15]$ = MMM 3, $\beta [16]$ = MMM 3, $\beta [17]$ = MMM 4, $\beta [18]$ = MMM 5, $\beta [19]$ = MMM 6, $\beta [20]$ = MMM 7, $\beta [21]$ = Time period 2010-2014, $\beta [22]$ = Time period 2015-2018, $\beta [23]$ = Time period 2019-2023

Posterior summaries from R2jags model output (the Meteropolis-Hastings Acccept-Reject steps counterpart) is given Table 9 below.

**Table 9:** R2jags posterior summary statistics for coefficients of a Hierarchical Logistic Regression Model: 3 chains, each with iter=10000; burnin=2000; thin=1; total draws=24000

Parameter	Mean	SD	2.5%	25%	50%	75%	97.5%	$\hat{\boldsymbol{R}}$	$\boldsymbol{n}_{\textbf{eff}}$
$\beta [1]$	-2.41	0.40	-3.21	-2.67	-2.40	-2.14	-1.65	1.00	6600
$\beta [2]$	0.11	0.05	0.01	0.08	0.12	0.15	0.22	1.00	2700
$\beta [3]$	-0.17	0.13	-0.42	-0.26	-0.17	-0.09	0.08	1.00	24000
$\beta [4]$	-0.38	0.12	-0.61	-0.46	-0.38	-0.30	-0.14	1.00	20000
$\beta [5]$	-0.84	0.12	-1.07	-0.92	-0.84	-0.76	-0.61	1.00	24000
$\beta [6]$	-1.16	0.12	-1.39	-1.24	-1.16	-1.08	-0.93	1.00	9200
$\beta [7]$	-2.40	0.13	-2.66	-2.49	-2.40	-2.31	-2.15	1.00	13000
$\beta [8]$	-5.38	0.36	-6.11	-5.62	-5.37	-5.14	-4.72	1.01	180
$\beta [9]$	1.75	0.22	1.32	1.61	1.75	1.90	2.17	1.00	24000
$\beta [10]$	-0.77	0.12	-1.00	-0.85	-0.77	-0.69	-0.54	1.00	5900
$\beta [11]$	-1.08	0.06	-1.20	-1.12	-1.08	-1.04	-0.96	1.00	24000
$\beta [12]$	1.12	0.35	0.47	0.88	1.10	1.34	1.85	1.00	3900
$\beta [13]$	-0.95	0.08	-1.11	-1.00	-0.94	-0.89	-0.79	1.00	1100
$\beta [14]$	-0.53	0.06	-0.64	-0.56	-0.53	-0.49	-0.41	1.00	7200
$\beta [15]$	0.18	0.10	-0.01	0.11	0.18	0.24	0.36	1.00	4400
$\beta [16]$	-0.07	0.13	-0.32	-0.15	-0.07	0.02	0.18	1.00	24000
$\beta [17]$	-0.08	0.16	-0.39	-0.18	-0.08	0.03	0.22	1.00	11000
$\beta [18]$	0.04	0.09	-0.13	-0.02	0.04	0.09	0.20	1.00	20000
$\beta [19]$	-0.14	0.15	-0.44	-0.24	-0.14	-0.04	0.14	1.00	7700
$\beta [20]$	-0.06	0.17	-0.39	-0.17	-0.05	0.06	0.27	1.00	4700
$\beta [21]$	-0.24	0.07	-0.39	-0.29	-0.24	-0.20	-0.10	1.00	3900
$\beta [22]$	-0.31	0.08	-0.46	-0.36	-0.31	-0.26	-0.16	1.00	24000
$\beta [23]$	-1.27	0.08	-1.43	-1.33	-1.27	-1.22	-1.12	1.00	1800

$\beta [1]$ = Intercept, $\beta [2]$ = Male, $\beta [3]$ = Age group $\ge$26-36, $\beta [4]$ = Age group $\ge$36-46, $\beta [5]$ = Age group $\ge$46-56, $\beta [6]$ = Age group $\ge$56-66, $\beta [7]$ = Age group $\ge$66-76, $\beta [8]$ = Age group 76+, $\beta [9]$ = Non-Indigenous, $\beta [10]$ = Lung diseases, $\beta [11]$ = Diabetes, $\beta [12]$ = CVD, $\beta [13]$ = Late referral, $\beta [14]$ = Obesity, $\beta [15]$ = MMM 3, $\beta [16]$ = MMM 3, $\beta [17]$ = MMM 4, $\beta [18]$ = MMM 5, $\beta [19]$ = MMM 6, $\beta [20]$ = MMM 7, $\beta [21]$ = Time period 2010-2014, $\beta [22]$ = Time period 2015-2018, $\beta [23]$ = Time period 2019-2023

Posterior summaries from MH model output from 3 chains, 8000 samples per chain (afer 2000 post-burnin, thinning =1, and a total of 24000 iterations) is given Table 10 below.

**Table 10:** MCMC summary for Metropolis-Hastings hierarchical logistic regression model convergence: 3 chains, 8000 samples per chain (post-burnin, thinned), total 24000 iterations

Parameter	Mean	SD	2.5%	25%	50%	75%	97.5%	$\hat{\boldsymbol{R}}$	$\boldsymbol{n}_{\textbf{eff}}$
Intercept	-2.33	0.37	-3.00	-2.60	-2.31	-2.06	-1.58	1.10	26
Male	0.13	0.06	0.02	0.10	0.13	0.17	0.24	1.01	1747
Age group $\ge$26–36	-0.16	0.13	-0.42	-0.25	-0.16	-0.07	0.08	1.02	434
Age group $\ge$36–46	-0.33	0.12	-0.57	-0.41	-0.33	-0.24	-0.10	1.02	307
Age group $\ge$46–56	-0.84	0.12	-1.08	-0.92	-0.84	-0.76	-0.62	1.02	345
Age group $\ge$56–66	-1.16	0.12	-1.39	-1.24	-1.16	-1.08	-0.94	1.02	320
Age group $\ge$66–76	-2.37	0.13	-2.63	-2.46	-2.37	-2.29	-2.12	1.02	454
Age group 76+	-5.26	0.34	-5.99	-5.48	-5.23	-5.02	-4.65	1.01	431
Non-Indigenous	1.77	0.28	1.23	1.57	1.79	1.97	2.26	1.18	49
Lung diseases	-0.81	0.12	-1.05	-0.89	-0.80	-0.72	-0.57	1.00	2316
Diabetes	-1.07	0.06	-1.20	-1.11	-1.07	-1.03	-0.95	1.00	3109
CVD	0.97	0.32	0.39	0.73	0.95	1.20	1.59	1.26	37
Late referral	-0.93	0.08	-1.09	-0.98	-0.93	-0.87	-0.77	1.00	3285
Obesity	-0.52	0.06	-0.64	-0.56	-0.52	-0.48	-0.41	1.00	3521
MMM 2	0.19	0.10	-0.01	0.13	0.19	0.26	0.39	1.00	1074
MMM 3	-0.02	0.13	-0.28	-0.11	-0.02	0.07	0.23	1.00	1911
MMM 4	-0.03	0.16	-0.35	-0.14	-0.03	0.07	0.27	1.00	1369
MMM 5	0.07	0.09	-0.10	0.01	0.07	0.13	0.24	1.00	952
MMM 6	-0.32	0.16	-0.65	-0.43	-0.32	-0.21	-0.02	1.01	1038
MMM 7	0.10	0.17	-0.24	-0.01	0.10	0.21	0.43	1.00	1126
Time period 2010–2014	-0.23	0.07	-0.37	-0.28	-0.23	-0.18	-0.09	1.00	1356
Time period 2015–2018	-0.29	0.08	-0.44	-0.34	-0.29	-0.24	-0.14	1.00	1267
Time period 2019–2023	-1.28	0.08	-1.44	-1.33	-1.28	-1.22	-1.12	1.00	1158

Posterior summaries from MH model output from 3 chains, 18000 samples per chain (afer 2000 post-burnin, thinning =1, and a total of 54000 iterations) is given Table 11 below.

**Table 11:** MCMC summary for Metropolis-Hastings hierarchical logistic regression model convergence: 3 chains, 18000 samples per chain (post-burnin, thinned), total 54000 iterations

Parameter	Mean	SD	2.5%	25%	50%	75%	97.5%	$\hat{\boldsymbol{R}}$	$\boldsymbol{n}_{\textbf{eff}}$
Intercept	-2.38	0.49	-3.49	-2.69	-2.36	-2.05	-1.48	32	1.082
Male	0.14	0.05	0.03	0.10	0.14	0.17	0.24	3449	1.001
Age group $\ge$26-36	-0.14	0.13	-0.41	-0.23	-0.14	-0.06	0.11	740	1.005
Age group $\ge$36-46	-0.31	0.12	-0.55	-0.39	-0.31	-0.23	-0.07	631	1.007
Age group $\ge$	-0.82	0.12	-1.06	-0.90	-0.82	-0.74	-0.58	597	1.007
Age group $\ge$	-1.14	0.12	-1.38	-1.22	-1.14	-1.06	-0.91	592	1.006
Age group $\ge$66-76	-2.35	0.13	-2.61	-2.44	-2.35	-2.26	-2.09	733	1.005
Age group $\ge$76+	-5.25	0.36	-6.02	-5.48	-5.24	-5.00	-4.59	1733	1.002
Non-Indigenous	1.73	0.24	1.27	1.56	1.73	1.90	2.20	121	1.104
Lung diseases	-0.81	0.12	-1.05	-0.89	-0.80	-0.72	-0.57	6765	1.000
Diabetes	-1.08	0.06	-1.20	-1.12	-1.07	-1.03	-0.95	6501	1.000
CVD	1.04	0.39	0.37	0.78	1.02	1.29	1.99	51	1.079
Late referral	-0.93	0.08	-1.09	-0.98	-0.93	-0.87	-0.76	7891	1.001
Obesity	-0.52	0.06	-0.64	-0.56	-0.52	-0.48	-0.40	7073	1.000
MMM 2	0.20	0.10	0.00	0.13	0.20	0.27	0.40	1955	1.001
MMM 3	-0.02	0.13	-0.27	-0.10	-0.02	0.07	0.24	4006	1.000
MMM 4	-0.02	0.16	-0.33	-0.13	-0.02	0.09	0.28	2874	1.001
MMM 5	0.08	0.09	-0.09	0.02	0.08	0.14	0.25	2022	1.000
MMM 6	-0.31	0.16	-0.63	-0.42	-0.31	-0.20	0.00	1948	1.001
MMM 7	0.11	0.17	-0.22	0.00	0.11	0.23	0.43	1401	1.001
Time group 2010-2014	-0.23	0.07	-0.38	-0.28	-0.23	-0.18	-0.09	2664	1.005
Time group 2015-2018	-0.29	0.08	-0.44	-0.34	-0.29	-0.24	-0.14	2472	1.005
Time group 2019-2023	-1.27	0.08	-1.44	-1.33	-1.27	-1.22	-1.11	2550	1.004

That is, 20000 iterations with 2000 burnings per chain

Summary of computational wall-clock time recorded by MH, R2jags and Stan is presented in Table 12.

**Table 12:** Comparison of computational time taken by the three approaches: Metropolis-Hastings (MH), R2jags and Stan

Approach	Hours	Minutes
MH		53.56
R2jags		54.41
Stan	2.0	33.29

### Shrinkage

The shrinkage analysis summarized in Table 13 presents empirical logits, shrunken logits, and several derived measures of shrinkage (pull toward the overall mean, distance moved, and percent change) for 34 centers with differing patient volumes. The analysis reveals clear patterns consistent with well-documented phenomena in hierarchical modelling, particularly the stabilising effect of partial pooling in the presence of varying sample sizes (centers with low versus high volume).

Across centers, shrinkage tends to pull the empirical logit estimates toward the group mean, with the direction and magnitude of adjustment driven largely by the degree of uncertainty around each center’s empirical estimate. For example, Center 1 (Medium volume) and Center 11 (Medium volume) have extremely low empirical logits (–3.76 and –6.91, respectively) that are adjusted substantially upward (toward the mean) in the shrunken logit (–2.38 and –2.49), corresponding to large negative distances moved and negative percent changes. This pattern reflects the classic behaviour of hierarchical models in the presence of sparse data: estimates from centers with fewer observations or rare events are “shrunk” more toward the overall mean to mitigate over-interpretation of noisy rates [55, 56].

In contrast, centers with large patient volume and more stable empirical logits, such as Center 2 (Large, 16.16% change) and Center 22 (Large, 16.37% change), often exhibit smaller adjustments. Some large centers even shift slightly away from the mean (positive “pull” values), reflecting the data’s strength in overriding the hierarchical prior to some extent. This is consistent with the principle that higher-volume groups yield more precise within-group estimates that require less shrinkage [57].

This volume–shrinkage relationship is a fundamental feature of hierarchical modelling: as sample size increases, the posterior for group effects is increasingly dominated by the group’s data rather than the group mean prior [58, 59]. In the extreme, with very large volume and rare outcome event rates, the shrinkage adjustment becomes minimal, reflecting strong within-center evidence.

“Distance moved” provides another lens, showing the raw change from empirical to shrunk logits. Large absolute distances (e.g., Center 11: -4.41) signal that the empirical estimate was quite unstable and the model relied heavily on partial pooling.

These findings manifest well-established results in hierarchical / small-area estimation. The shrinkage phenomenon observed aligns with the seminal work of Efron and Morris (1975), who demonstrated that in the presence of many groups with varying sample sizes, hierarchical models that perform partial pooling often yield estimates with lower overall mean squared error compared to separate (non-hierarchical) estimates [59]. Gelman et al. (2006) and Raudenbush and Bryk (2002) further articulate how hierarchical Bayesian models adaptively balance within-group information and between-group variance, particularly when group sample sizes differ substantially [55, 58].

More recent applications in healthcare quality assessment reflect similar shrinkage patterns, where low-volume providers’ performance metrics are “shrunk” more strongly toward the overall mean to avoid over-interpretation of random noise [60, 61]. Such adjustments are particularly critical in binary outcome models, where rare events (e.g., adverse events) can lead to extreme empirical logits that do not reflect reliable signals without shrinkage.

Practically, this shrinkage analysis suggests that care should be taken when interpreting raw empirical performance metrics for centers with small or moderate patient volumes. Without partial pooling, extreme empirical logits at low-volume centers may be misinterpreted as true effects rather than sampling variation. The hierarchical shrinkage approach stabilises these estimates, producing more robust and conservative metrics that better reflect the true underlying center performance.

This pattern supports policy and clinical decision frameworks that favour hierarchical or empirical Bayes adjustments in performance reporting, as discussed in health services research [60, 61].

**Table 13:** Empirical and shrinkage logits by center

Center	ID	Empirical logit	Shrunken logit	Pull toward mean	Distance moved	% change	Volume category
1	1	-3.76	-2.38	-0.12	-1.38	-36.75	Medium (50–100)
2	2	-2.12	-2.46	-0.08	0.34	16.16	Large (>100)
3	3	-4.46	-2.92	-0.03	-1.54	-34.58	Large (>100)
4	4	-1.92	-2.47	-0.08	0.55	28.88	Large (>100)
5	5	-4.06	-2.66	-0.05	-1.41	-34.57	Large (>100)
6	6	-1.92	-2.40	-0.11	0.48	24.72	Large (>100)
7	7	-2.84	-1.51	0.03	-1.33	-46.88	Large (>100)
8	8	-1.28	-1.45	0.03	0.17	13.33	Large (>100)
9	9	-4.23	-2.44	-0.09	-1.79	-42.27	Large (>100)
10	10	-2.29	-2.66	-0.05	0.37	16.05	Large (>100)
11	11	-6.91	-2.49	-0.07	-4.41	-63.88	Medium (50–100)
12	12	-2.26	-2.56	-0.06	0.30	13.18	Large (>100)
13	13	-4.26	-2.59	-0.05	-1.67	-39.17	Medium (50–100)
14	14	-1.89	-2.26	-0.39	0.37	19.32	Large (>100)
15	15	-3.26	-1.92	0.08	-1.33	-40.97	Medium (50–100)
16	16	-1.98	-2.10	0.21	0.12	6.29	Large (>100)
17	17	-6.91	-2.77	-0.04	-4.14	-59.96	Medium (50–100)
18	18	-1.95	-2.21	-5.05	0.26	13.08	Large (>100)
19	19	-3.20	-1.60	0.03	-1.60	-50.00	Large (>100)
20	20	-1.47	-1.51	0.03	0.04	2.90	Large (>100)
21	21	-4.14	-1.91	0.07	-2.23	-53.79	Large (>100)
22	22	-1.83	-2.13	0.29	0.30	16.37	Large (>100)
23	23	-3.66	-1.93	0.08	-1.73	-47.27	Medium (50–100)
24	24	-1.64	-2.27	-0.32	0.63	38.67	Large (>100)
25	25	-4.51	-2.57	-0.06	-1.95	-43.12	Large (>100)
26	26	-2.59	-2.63	-0.05	0.04	1.69	Small (10–50)
27	27	-3.89	-2.12	0.26	-1.77	-45.50	Small (10–50)
28	28	-2.33	-2.49	-0.07	0.16	7.08	Large (>100)
29	29	-2.45	-1.49	0.03	-0.96	-39.16	Medium (50–100)
30	30	-2.10	-2.27	-0.33	0.17	7.88	Large (>100)
31	31	-2.90	-1.36	0.03	-1.54	-53.08	Large (>100)
32	32	-1.71	-1.41	0.03	-0.30	-17.46	Large (>100)
33	33	-2.74	-2.16	0.51	-0.58	-21.12	Small (10–50)
34	34	-1.49	-2.09	0.19	0.60	40.24	Large (>100)

## Appendix B: A step-by-step derivation of both results in the Logistic Derivatives lemma

In the below, we presented the derivative of the Logistic Function

- Given the logistic function:
  $$\begin{aligned} \sigma (\eta ) = \frac{1}{1 + e^{-\eta }} = (1 + e^{-\eta })^{-1} \end{aligned}$$
- Step 1: Rewrite using exponent rules
  $$\begin{aligned} \sigma (\eta ) = (1 + e^{-\eta })^{-1} \end{aligned}$$
- Step 2: Apply chain rule for differentiation
  $$\begin{aligned} \frac{d\sigma }{d\eta } = -1(1 + e^{-\eta })^{-2} \cdot \frac{d}{d\eta }(1 + e^{-\eta }) \end{aligned}$$
- Step 3: Differentiate the inner term
  $$\begin{aligned} \frac{d}{d\eta }(1 + e^{-\eta }) = 0 + (-e^{-\eta }) = -e^{-\eta } \end{aligned}$$
- Step 4: Combine results
  $$\begin{aligned} \frac{d\sigma }{d\eta } = -1(1 + e^{-\eta })^{-2} \cdot (-e^{-\eta }) = \frac{e^{-\eta }}{(1 + e^{-\eta })^2} \end{aligned}$$
- Step 5: Express in terms of $\sigma (\eta )$
  $$\begin{aligned} = \frac{1}{1 + e^{-\eta }} \cdot \frac{e^{-\eta }}{1 + e^{-\eta }} = \sigma (\eta ) \cdot \left( \frac{1 + e^{-\eta } - 1}{1 + e^{-\eta }}\right) \end{aligned}$$
  $$\begin{aligned} = \sigma (\eta ) \left( 1 - \frac{1}{1 + e^{-\eta }}\right) = \sigma (\eta )(1 - \sigma (\eta )) \end{aligned}$$

## Appendix C: Normal-inverse-gamma conjugacy

A step-by-step derivation and proof of the Normal-Inverse-Gamma conjugacy is shown below:

### Proof

**Step 1:** Specify the prior and likelihood

The Inverse-Gamma (IG) prior for $\sigma _u^2$:

$$\begin{aligned} P(\sigma _u^2) = \frac{b_0^{a_0}}{\Gamma (a_0)} (\sigma _u^2)^{-(a_0 + 1)} e^{-b_0/\sigma _u^2} \end{aligned}$$

The normal likelihood for $u_j$ given $\sigma _u^2$:

$$\begin{aligned} P(\{u_j\}|\sigma _u^2) = \prod _{j=1}^J \frac{1}{\sqrt{2\pi \sigma _u^2}} \exp \left( -\frac{u_j^2}{2\sigma _u^2}\right) \end{aligned}$$

**Step 2:** Write the joint distribution

$$\begin{aligned} & P(\{u_j\}, \sigma _u^2) = P(\{u_j\}|\sigma _u^2)P(\sigma _u^2)\\ & = \left[ (2\pi \sigma _u^2)^{-J/2} \exp \left( -\frac{1}{2\sigma _u^2}\sum \limits _{j=1}^J u_j^2\right) \right] \\ & \quad \times \left[ \frac{b_0^{a_0}}{\Gamma (a_0)} (\sigma _u^2)^{-(a_0 + 1)} e^{-b_0/\sigma _u^2}\right] \end{aligned}$$

**Step 3:** Combine terms and simplify

$$\begin{aligned} \propto (\sigma _u^2)^{-J/2} (\sigma _u^2)^{-(a_0 + 1)} \exp \left[ -\frac{1}{\sigma _u^2}\left( b_0 + \frac{1}{2}\sum \limits _{j=1}^J u_j^2\right) \right] \end{aligned}$$

$$\begin{aligned} = (\sigma _u^2)^{-(a_0 + J/2 + 1)} \exp \left[ -\frac{1}{\sigma _u^2}\left( b_0 + \frac{1}{2}\sum \limits _{j=1}^J u_j^2\right) \right] \end{aligned}$$

**Step 4:** Recognize the Inverse-Gamma form Comparing with the IG density form:

$$\begin{aligned} \text {IG}(x|a,b) \propto x^{-(a+1)} e^{-b/x} \end{aligned}$$

we identify:

$$\begin{aligned} a & = a_0 + \frac{J}{2} \\ b & = b_0 + \frac{1}{2}\sum \limits _{j=1}^J u_j^2 \end{aligned}$$

It can be concluded that the posterior distribution for $\sigma ^2_u$ is:

$$\begin{aligned} \sigma _u^2 \mid \{u_j\} \sim \text {IG}\left( a_0 + \frac{J}{2}, b_0 + \frac{1}{2}\sum \limits _{j=1}^J u_j^2\right) \end{aligned}$$

$$\square $$

## Appendix D: Computing Log Standardised Incidence Ration (Log-SIR)

10. **Initialization**:
  - Pre-compute $Y_j$ (observed counts) and $N_j$ (patient counts)
  - Load $\hat{p}_{j[i]}$ posterior samples (matrix: patients $\times$ iterations)
11. **For each MCMC iteration** *s* :
  12. Sample $$\pi _j^{(s)} \sim \text {Beta}(a + Y_j, b + N_j - Y_j)$$
  13. Extract $\hat{p}_{j[i]}^{(s)}$ (column *s* of p_hat_matrix)
  14. Compute $$E_j^{(s)} = \sum \nolimits _i \hat{p}_{j[i]}^{(s)} \rightarrow $$ expected_samples[s,j]
  15. Calculate $$\log (\text {SIR}_j^{(s)}) \rightarrow $$ log_sir_samples[s,j]
16. **Output**:
  - Posterior samples for $\pi _j$, $E_j$, and $\log (\text {SIR}_j)$

## Appendix E: Gibbs sampler via MH algorithm

### Metropolis-Hastings update for $\alpha$

We aim to sample from the posterior distribution of the intercept parameter $\alpha$:

$$\begin{aligned} p(\alpha \mid \cdot ) \propto \left[ \prod _{i=1}^n p_i^{Y_i} (1 - p_i)^{1 - Y_i}\right] \cdot \exp \left( -\frac{\alpha ^2}{2\sigma _\alpha ^2}\right) \end{aligned}$$

where:

### MH algorithm for $\alpha$

17. **Propose:**
  $$\begin{aligned} \alpha ^* \sim \mathcal {N}(\alpha , \tau _\alpha ^2) \end{aligned}$$
18. **Compute predicted probabilities under current and proposed** $\boldsymbol{\alpha }$**:**
  $$\begin{aligned} \eta _i = \alpha + X_i^\top \beta + u_{j[i]}, \quad p_i = \frac{e^{\eta _i}}{1 + e^{\eta _i}} \end{aligned}$$
  $$\begin{aligned} \eta _i^* = \alpha ^* + X_i^\top \beta + u_{j[i]}, \quad p_i^* = \frac{e^{\eta _i^*}}{1 + e^{\eta _i^*}} \end{aligned}$$
19. **Compute log-likelihoods:**
  $$\begin{aligned} \log L(\alpha ) = \sum \limits _{i=1}^n \left[ Y_i \log (p_i) + (1 - Y_i) \log (1 - p_i) \right] \end{aligned}$$
  $$\begin{aligned} \log L(\alpha ^*) = \sum \limits _{i=1}^n \left[ Y_i \log (p_i^*) + (1 - Y_i) \log (1 - p_i^*) \right] \end{aligned}$$
20. **Compute log-prior terms:**
  $$\begin{aligned} \log p(\alpha ) = -\frac{\alpha ^2}{2\sigma _\alpha ^2}, \quad \log p(\alpha ^*) = -\frac{(\alpha ^*)^2}{2\sigma _\alpha ^2} \end{aligned}$$
21. **Compute log-posterior difference:**
  $$\begin{aligned} \Delta = \log L(\alpha ^*) - \log L(\alpha ) + \log p(\alpha ^*) - \log p(\alpha ) \end{aligned}$$
22. **Accept with probability:**
  $$\begin{aligned} \min \left( 1, \exp (\Delta )\right) \end{aligned}$$

### Metropolis-Hastings update for $\beta _k$

We now derive the Metropolis-Hastings update for a single regression coefficient $\beta _k$:

$$\begin{aligned} p(\beta _k \mid \cdot ) \propto \left[ \prod _{i=1}^n p_i^{Y_i} (1 - p_i)^{1 - Y_i}\right] \cdot \exp \left( -\frac{\beta _k^2}{2\sigma _\beta ^2}\right) \end{aligned}$$

#### MH algorithm for $\beta _k$

23. **Propose:**
  $$\begin{aligned} \beta _k^* \sim \mathcal {N}(\beta _k, \tau _\beta ^2) \end{aligned}$$
24. **Compute predicted probabilities:** Let $X_i^\top \beta ^{(-k)}$ be the linear predictor excluding $\beta _k X_{ik}$. Then:
  $$\begin{aligned} \eta _i = \alpha + X_i^\top \beta + u_{j[i]} = \alpha + \sum \limits _{r \ne k} X_{ir} \beta _r + X_{ik} \beta _k + u_{j[i]} \end{aligned}$$
  $$\begin{aligned} \eta _i^* = \alpha + \sum \limits _{r \ne k} X_{ir} \beta _r + X_{ik} \beta _k^* + u_{j[i]} \end{aligned}$$
  $$\begin{aligned} p_i = \frac{e^{\eta _i}}{1 + e^{\eta _i}}, \quad p_i^* = \frac{e^{\eta _i^*}}{1 + e^{\eta _i^*}} \end{aligned}$$
25. **Compute log-likelihoods:**
  $$\begin{aligned} \log L(\beta _k) = \sum \limits _{i=1}^n \left[ Y_i \log (p_i) + (1 - Y_i) \log (1 - p_i) \right] \end{aligned}$$
  $$\begin{aligned} \log L(\beta _k^*) = \sum \limits _{i=1}^n \left[ Y_i \log (p_i^*) + (1 - Y_i) \log (1 - p_i^*) \right] \end{aligned}$$
26. **Compute log-prior terms:**
  $$\begin{aligned} \log p(\beta _k) = -\frac{\beta _k^2}{2\sigma _\beta ^2}, \quad \log p(\beta _k^*) = -\frac{(\beta _k^*)^2}{2\sigma _\beta ^2} \end{aligned}$$
27. **Compute log-posterior difference:**
  $$\begin{aligned} \Delta = \log L(\beta _k^*) - \log L(\beta _k) + \log p(\beta _k^*) - \log p(\beta _k) \end{aligned}$$
28. **Accept with probability:**
  $$\begin{aligned} \min \left( 1, \exp (\Delta )\right) \end{aligned}$$

### Summary of MH accept-reject rule

For both $\alpha$ and $\beta _k$:

- Propose a new value from a symmetric normal proposal.
- Compute the log-likelihood and log-prior under both current and proposed values.
- Take the difference of log-posterior values and exponentiate to get the MH acceptance ratio.
- Accept the proposed value with probability:
  $$\begin{aligned} \min \left( 1, \exp (\text {log-posterior difference})\right) \end{aligned}$$

### Metropolis-Hastings update for $u_j$

#### MH algorithm for $u_j$

We aim to update the random effect $u_j$ using a Metropolis-Hastings (MH) algorithm. The full conditional posterior of $u_j$ is proportional to:

$$\begin{aligned} p(u_j \mid \cdot ) \propto \left[ \prod _{i \in \mathcal {I}_j} p_i^{Y_i} (1 - p_i)^{1 - Y_i}\right] \cdot \exp \left( -\frac{u_j^2}{2\sigma _u^2}\right) \end{aligned}$$

where:

- $\mathcal {I}_j$ is the set of patients in hospital *j*
- $$\eta _i = \alpha + X_i^\top \beta + u_j$$ for $$i \in \mathcal {I}_j$$

#### Metropolis-Hastings algorithm

29. **Propose:**
  $$\begin{aligned} u_j^* \sim \mathcal {N}(u_j, \tau _u^2) \end{aligned}$$
30. **Compute the log-likelihood under current and proposed values:** For the current $u_j$, compute
  $$\begin{aligned} \log L_j(u_j) = \sum \limits _{i \in \mathcal {I}_j} \left[ Y_i \log (p_i) + (1 - Y_i) \log (1 - p_i) \right] \end{aligned}$$
  where
  $$\begin{aligned} \eta _i = \alpha + X_i^\top \beta + u_j, \quad p_i = \frac{e^{\eta _i}}{1 + e^{\eta _i}} \end{aligned}$$
  Similarly, for the proposed $u_j^*$:
  $$\begin{aligned} \log L_j(u_j^*) = \sum \limits _{i \in \mathcal {I}_j} \left[ Y_i \log (p_i^*) + (1 - Y_i) \log (1 - p_i^*) \right] \end{aligned}$$
  where
  $$\begin{aligned} \eta _i^* = \alpha + X_i^\top \beta + u_j^*, \quad p_i^* = \frac{e^{\eta _i^*}}{1 + e^{\eta _i^*}} \end{aligned}$$
31. **Compute log-prior terms:**
  Assuming a normal prior:
  $$\begin{aligned} u_j \sim \mathcal {N}(0, \sigma _u^2) \Rightarrow \log p(u_j) = -\frac{u_j^2}{2\sigma _u^2} \end{aligned}$$
  $$\begin{aligned} \log p(u_j^*) = -\frac{(u_j^*)^2}{2\sigma _u^2} \end{aligned}$$
32. **Compute log-posterior difference:**
  $$\begin{aligned} \Delta = \log L_j(u_j^*) - \log L_j(u_j) + \log p(u_j^*) - \log p(u_j) \end{aligned}$$
33. **Accept with probability:**
  $$\begin{aligned} \min \left( 1, \exp (\Delta )\right) \end{aligned}$$

in summary, to update $u_j$, accept the proposed value $u_j^*$ with probability:

$$\begin{aligned} \min \left( 1, \exp (\log L_j(u_j^*) - \log L_j(u_j) + \log p(u_j^*) - \log p(u_j))\right) \end{aligned}$$

## Appendix F: R codes: Gibbs sampler via Metropolis-Hastings accept- reject steps

We will share the R codes used for running Gibbs sampler via Metropolis-Hastings Accept- Reject Steps up on acceptance of our manuscript for publication.

## Code availability

We will share the R codes used for running Gibbs sampler via Metropolis-Hastings Accept-Reject Steps upon acceptance of our manuscript for publication.
